# Bacterial Communities in Women with Bacterial Vaginosis: High Resolution Phylogenetic Analyses Reveal Relationships of Microbiota to Clinical Criteria

**DOI:** 10.1371/journal.pone.0037818

**Published:** 2012-06-18

**Authors:** Sujatha Srinivasan, Noah G. Hoffman, Martin T. Morgan, Frederick A. Matsen, Tina L. Fiedler, Robert W. Hall, Frederick J. Ross, Connor O. McCoy, Roger Bumgarner, Jeanne M. Marrazzo, David N. Fredricks

**Affiliations:** 1 Vaccine & Infectious Disease Division, Fred Hutchinson Cancer Research Center, Seattle, Washington, United States of America; 2 Department of Laboratory Medicine, University of Washington, Seattle, Washington, United States of America; 3 Public Health Science Division, Fred Hutchinson Cancer Research Center, Seattle, Washington, United States of America; 4 Department of Microbiology, University of Washington, Seattle, Washington, United States of America; 5 Department of Medicine, University of Washington, Seattle, Washington, United States of America; Columbia University, United States of America

## Abstract

**Background:**

Bacterial vaginosis (BV) is a common condition that is associated with numerous adverse health outcomes and is characterized by poorly understood changes in the vaginal microbiota. We sought to describe the composition and diversity of the vaginal bacterial biota in women with BV using deep sequencing of the 16S rRNA gene coupled with species-level taxonomic identification. We investigated the associations between the presence of individual bacterial species and clinical diagnostic characteristics of BV.

**Methodology/Principal Findings:**

Broad-range 16S rRNA gene PCR and pyrosequencing were performed on vaginal swabs from 220 women with and without BV. BV was assessed by Amsel’s clinical criteria and confirmed by Gram stain. Taxonomic classification was performed using phylogenetic placement tools that assigned 99% of query sequence reads to the species level. Women with BV had heterogeneous vaginal bacterial communities that were usually not dominated by a single taxon. In the absence of BV, vaginal bacterial communities were dominated by either *Lactobacillus crispatus* or *Lactobacillus iners*. *Leptotrichia amnionii* and *Eggerthella* sp. were the only two BV-associated bacteria (BVABs) significantly associated with each of the four Amsel’s criteria. Co-occurrence analysis revealed the presence of several sub-groups of BVABs suggesting metabolic co-dependencies. Greater abundance of several BVABs was observed in Black women without BV.

**Conclusions/Significance:**

The human vaginal bacterial biota is heterogeneous and marked by greater species richness and diversity in women with BV; no species is universally present. Different bacterial species have different associations with the four clinical criteria, which may account for discrepancies often observed between Amsel and Nugent (Gram stain) diagnostic criteria. Several BVABs exhibited race-dependent prevalence when analyzed in separate groups by BV status which may contribute to increased incidence of BV in Black women. Tools developed in this project can be used to study microbial ecology in diverse settings at high resolution.

## Introduction

The microbiota of the human vagina can significantly impact the health of women, their fetuses and newborn infants [1], [2], [3]. Bacterial vaginosis (BV) is a highly prevalent condition, affecting 29% of reproductive age women in the United States [4]. BV is associated with increased risks for pelvic inflammatory disease [5], preterm birth [6], and acquisition of HIV [7]. BV can be diagnosed using Amsel’s clinical criteria comprising a set of four characteristics including vaginal discharge, amine odor, elevated pH, and the presence of clue cells, wherein 3 of 4 criteria must be present to make a positive diagnosis [8]. The gold standard for diagnosis of BV in research settings is analysis of vaginal fluid Gram stains [9]. About half the time, women with BV are asymptomatic and it is unclear what factors promote symptoms in some women.

Cultivation and molecular studies have shown that BV is characterized by an overgrowth of diverse anaerobes and a loss of most *Lactobacillus* species [10]. While BV can be treated with antibiotics including metronidazole and clindamycin [11], relapse rates are high and factors leading to relapse are poorly understood. Moreover, the pathogenesis of BV is unclear. A critical first step in comprehending this condition is to intensively catalog the microbial species and community profiles associated with BV. Few studies to date have described the diversity of microbial communities in this condition using both deep sequencing methods and species-level taxonomic classification. The ability to distinguish between bacterial species is important as different species are known to have distinct biological functions and clinical impacts. Most deep sequencing studies examining the microbiota associated with BV have samples from relatively few women [12], [13], [14] or have not documented concordant clinical data [13], [14]. As a result, high-throughput sequencing studies have not systematically assessed the association of vaginal bacterial species with clinical characteristics.

Many commonly used bioinformatics tools and databases do not accurately classify bacteria to the species level when using high-throughput sequencing of 16S rRNA gene amplicons. This is particularly problematic in the human vagina where there are several novel *Clostridiales* bacteria that are biologically important but not resolved using existing online tools such as the RDP [15] or Greengenes [16]. One study of the vaginal microbiota in reproductive age women identified lactobacilli to species level, but did not do the same for BV-associated bacteria [17]. The public databases also lack some taxa that are important for the understanding of bacterial vaginosis. For example, BV-associated bacterium-1 (BVAB-1), BVAB-2 and BVAB-3 are novel bacteria in the *Clostridiales* Order that are highly specific for BV [18].

In this cross-sectional study, we sought to comprehensively describe the bacterial census and community structures found in women with BV using high-throughput sequencing and species-level classification. For this purpose, we built a bioinformatics pipeline that relies on the placement of query sequences on a phylogenetic tree constructed with vaginal niche-specific reference sequences. Species-level classification was enabled by the combination of novel computational tools and extensive curation of a vaginal specific “reference package” containing sequences, taxonomic information and a phylogenetic tree. Using the species-level classification outputs, we first investigated whether a core microbiome is present in women with BV, and examined how these bacterial communities differ from women without BV. Second, the refined classification output allowed us to determine if novel species can be detected in the human vagina using deep sequencing. Third, we hypothesized that bacterial community sub-types may be shaped by synergistic or antagonistic relationships among individual BV-associated bacteria. Hence, we examined bacterial co-occurrence and whether there are distinct sub-types of BV-associated bacterial community profiles. Fourth, we explored whether the presence of particular bacterial species or community types was different in Black women with and without BV when compared with White women. Previous studies of asymptomatic women have suggested that women of different racial groups have different community profiles [17]. However, Black women also have higher rates of BV, and therefore it is unclear if Black women still have different community types when controlling for BV status. Fifth, we determined the associations between vaginal bacterial community composition and clinical diagnostic features (Amsel’s criteria) in order to assess the impact of individual members of the microbiota on these features.

## Materials and Methods

### Study Population and Sample Collection

This cross-sectional study enrolled 242 women from the Public Health, Seattle and King County Sexually Transmitted Diseases Clinic (STD clinic) between September 2006 and June 2010. The study was approved by the Institutional Review Board at the Fred Hutchinson Cancer Research Center. All study participants provided written informed consent. Participants were interviewed regarding medical history and sexual behaviors, and underwent a standard physical examination including a pelvic examination with speculum for collection of samples. Swabs of vaginal fluid were collected for Gram staining, pH, saline microscopy and potassium hydroxide preparation. Samples for DNA extraction and PCR were obtained using polyurethane foam swabs (Epicentre Biotechnologies, Madison, WI) that were brushed against the lateral vaginal wall, re-sheathed and frozen immediately at −20°C and subsequently held at −80°C. BV was diagnosed using Amsel’s criteria [8] and confirmed by Gram stains [9]. Subjects with BV were treated with 5 g 0.75% intravaginal metronidazole gel used each night for 5 days. Testing for sexually transmitted infections and other vaginal infections was performed as previously described [19].

### DNA Extraction, DNA Quantification, and qPCR

DNA was extracted from swabs using the Ultra Clean Soil DNA Kit or the similar Bacteremia Kit (Mobio, Carlsbad, CA), eluted in 150 µl buffer and diluted 1∶1 in 1 mM Tris, 0.1 mM EDTA buffer. Sham digests from swabs without human contact were used to assess contamination from extraction reagents or collection swabs. Total bacterial DNA concentration (16S rRNA gene copies) in each sample were measured using a quantitative PCR (qPCR) assay developed for this study based on TaqMan chemistry with broad-range 16S rRNA gene primers (338F 5′-ACTCCTRCGGGAGGCAGCAG-3′, 806R 5′-GGACTACCVGGGTATCTAAT-3′) and a hydrolysis probe (5′-FAM-TKACCGCGGCTGCTGGCAC-TAMRA-3′). Core reagents were from Applied Biosystems (Carlsbad, CA) and master mixes contained buffer A (1X), deoxynucleotide triphosphates (1 mM), magnesium (3 mM), AmpErase uracil-N-glycosylase (0.05 U) and AmpliTaq Gold LD polymerase (2.2 U) per reaction. The forward primer was added at 0.8 µM per reaction and the reverse primer at 1 µM. The final probe concentration was 200 µM. All reagents except water were filtered to minimize contamination using a Microcon YM-100 centrifugal filter unit (Millipore, Billerica, MA) at 2000 rpm for 35 min, 5000 rpm for 5 min and finally, 8000 rpm for 5 min. Water (MoBio, Carlsbad CA) was filtered using an Amicon Ultra-15 centrifugal filter unit (Millipore, Billerica, MA). Two µl of 1∶1 diluted DNA was used in each qPCR assay. Reactions underwent 42 cycles of amplification on the Applied Biosystems 7500 real-time cycler with a 95°C melt for 15 seconds, 55°C anneal for 39 s, and 72°C extension for 30 s. *Escherichia coli* plasmid standards were run for each reaction ranging from 10^7^ to 10 gene copies; values are reported as 16S rRNA gene copies/swab.

### Pyrosequencing of Barcoded 16S rRNA Gene Amplicons from Vaginal Samples

Broad-range PCR amplification of the V3–V4 hypervariable regions [20] of the 16S rRNA gene was performed using a 338F primer formulation [21] targeting the domain bacteria, including the *Plancomycetales* and the *Verrucomicrobiales,* along with 806R reverse primer with 8-bp barcodes to facilitate multiplexing. The forward primers used were: FusA338F1 GCCTCCCTCGCGCCATCAG *GC* **ACTCCTRCGGGAGGCAGCAG**, FusA 338F2 GCCTCCCTCGCGCCATCAG *GC* **ACACCTACGGGTGGCTGC** and FusA338F3 GCCTCCCTCGCGCCATCAG *GC* **ACACCTACGGGTGGCAGC** whose formulation was made at a ratio of 3∶1∶1. The reverse primer FusB806R was GCCTTGCCAGCCCGCTCAGNNNNNNNN*GC* **GGACTACCVGGGTATCTAAT**. The underlined sequence represents the 454 Life Sciences FLX sequencing primers FusA and FusB in 338 F and 806R primers respectively, and the italicized GC was used as a linker sequence to improve PCR efficiency. The N’s in the reverse primer are barcodes [22] and the bold letters denote the primer sequences targeting the 16S rRNA gene. Primers used are displayed in Table S1. DNA quantities ranging from 9×10^4^−3.5×10^6^ bacterial 16S rRNA gene copies from each vaginal sample were added to the PCR. Samples with fewer than 9×10^4^ gene copies per 2 µl were excluded for pyrosequencing as they yielded fewer than 500 sequence reads per sample. This led to the exclusion of 22 samples resulting in a final study population of 220 women. Master mixes contained *Pfu* reaction Buffer (1X), deoxynucleotide triphosphates (0.2 mM), forward primer (0.2 µM), reverse primer (0.4 µM) and *PfuTurbo* Hotstart DNA polymerase (1.5 U) for each PCR reaction. PCR cycling conditions included a polymerase activation step at 95°C for 2 min, followed by 30 cycles of 95°C for 30 s (denaturing), 55°C for 40 s (annealing) and 72°C for 1 min (extension). A final extension step of 72°C for 10 min completed the PCR. PCR reactions were prepared in duplicate for every sample and amplicons were visualized on 1.5% agarose gels with GelRed staining (Biotium, Hayward CA). The duplicate reactions were combined and every sample was processed through a two part clean-up to remove amplification primers and reaction buffers. The first step included filter purification using 0.5 mL Amicon Ultra centrifugal filter units (100 K) (Millipore, Billerica, MA) to reduce non-specific low molecular weight amplification products, followed by a second purification using AMPure beads (Agencourt). Each sample was quantified using the Quant-iT PicoGreen dsDNA HS assay (Invitrogen) and equimolar quantities were pooled. Emulsion PCR of the amplicon pools and 454 FLX pyrosequencing using 454 Life Sciences primer B (targeting the V4 hypervariable region of the 16S rRNA gene) was performed according to the manufacturer’s instructions. Negative controls included sham digests that were processed the same way as vaginal swabs to assess contamination from DNA extraction or PCR reagents.

### Pre-processing of Raw Sequence Reads

Sequences were pre-processed to assess overall quality, classify by barcodes, trim barcodes and primer sequences, and remove low quality reads. Quality assessment criteria were established following initial inspection. To be included in subsequent analyses, reads had to: (1) start with a known barcode; (2) contain the exact sequence of the 16S rRNA gene portion of the FusB806R primer; (3) consist of at least 200 nucleotides, in addition to barcode and primer; and (4) have average per-base -log_10_ quality score 35 or more. Reads were clipped to retain high quality spans with 90% Phred base quality scores greater than 15 in 30 mer windows. Pre-processing facilities are available in the R/Bioconductor package *microbiome*. The package creates a short quality assessment report highlighting important aspects of the raw data, and allows read selection for subsequent analysis.

### Vaginal Microbiome-specific Reference Sequences

A set of 16S rRNA gene reference sequences representing bacterial taxa associated with the vaginal mucosa was assembled, aligned, and used to create a phylogenetic tree. A preliminary list of taxa was compiled based on our previous studies [18], [23] and reports in the literature [24], [25]. We iteratively added additional taxa to this list during several rounds of classification. Candidate sequences representing each taxon were downloaded from the RDP website [15] or included from our in-house database of vaginal bacterial sequences generated by conventional Sanger sequencing methods from cloned bacterial PCR products [18], [23]. Sequences obtained from the RDP were labeled using the provided NCBI taxonomic annotation. Locally generated sequences were assigned to NCBI taxa by inspection and phylogenetic analysis. The NCBI taxonomy was extended to include provisional taxa to accommodate sequences that could not be otherwise assigned, such as bacterial vaginosis associated bacterium 1 (BVAB1), BVAB2, and BVAB3 (see Table S2 for a tally of sequences representing each taxon); lineages are defined in the vaginal-16S rRNA-reference package (see below). We found that sequences in public repositories were frequently assigned to the incorrect taxon. Potentially mislabeled sequences were identified by calculating pairwise distances (i.e., the fraction of non-identical nucleotides) among all sequences belonging to a given taxon. For each taxon, a primary reference sequence “S” with the smallest median distance to all other sequences was identified; sequences with a distance to “S” greater than 0.015 were discarded. The cutoff was chosen empirically, but corresponds to a threshold of 98.5% identity, which is similar to thresholds used for species-level taxonomic assignment described in the literature [26]. A collection of sequences was selected from those remaining by first retaining the primary reference sequence “S” and any sequences originating from reference or type strains; additional sequences were added to the collection to maximize the sum of pairwise distances among those selected. In most cases, N = 5 sequences were selected to represent each taxon; in some cases fewer sequences were selected to represent taxa of minimal biological importance (e.g., *Pseudomonas* spp.) or more were selected to improve species-level resolution (e. g., *Lactobacillus* spp.). Sequences representing additional species were added to the reference set iteratively as follows: after sequence alignment, and construction of a phylogenetic tree (described below), we used the resulting reference set to perform classification. We used usearch v5.0 [27] to compare reads that could not be classified to the species level to full-length 16S rRNA gene sequences downloaded from the RDP. Species represented by similar matches were added to the original list of taxa, and the reference set was recompiled. This process was repeated until >99% of reads were classified to the species level. The resulting set of 796 reference sequences were aligned using cmalign 1.0.2 [28] according to a 16S rRNA alignment profile provided by the RDP [29]. A maximum-likelihood phylogenetic tree was inferred with RAxML 7.2.7 using the General Time Reversible model of nucleotide substitution [30], [31]. A sequence alignment and phylogenetic tree representing the vaginal reference sequences spanning the V3/V4 regions are provided in the phylogenetic Reference Package S1 (vaginal-0.9.2-raxml.refpkg).

### Taxonomic Classification of Sequence Reads

454 pyrosequencing reads (“query” sequences) were aligned with cmalign 1.0.2 [28]. The resulting alignments were merged with reference alignments (using ‘cmalign –merge’) to place all sequences in the same alignment register. Query sequences were then placed on a phylogenetic tree of reference sequences using *pplacer v1.1* [32]. *pplacer* finds the optimal insertion of reads into a fixed phylogenetic tree according to the maximum-likelihood or Bayesian posterior probability criteria. The program was run using posterior probability mode (using the –p and –informative-prior flags), which defines an informative prior for pendant branch lengths with a mean derived from the average distances from the edge in question to the leaves of the tree. *pplacer* infers taxonomic classifications of a collection of reads by associating edges of a phylogenetic tree with taxonomic labels. It does so for a given edge by finding the most specific taxonomic rank such that there is exactly one taxonomic identifier at that rank which is present on one or more leaves on either side of the edge. These taxonomic edge labels are used to infer taxonomic classifications for a given read by assigning the posterior probability of a taxonomic classification to be the sum of the posterior probabilities of the edges with that taxonomic label. Classifications were then collated with the “guppy classify” command (see below) using a posterior probability cutoff of 0.9. To provide more specific classification results, for each genus-level or higher classification, a compound name (eg: “*Streptococcus mitis/oralis*”) was constructed using up to three taxonomic names each with an individual likelihood >0.05 at a more specific rank. These labels did not contribute significantly to the overall results as only 0.25% classified reads were named in this way.

### Classifier Validation

Taxonomic assignment for *Lactobacillus* spp. was systematically evaluated by assembling a validation set of 16S rRNA gene sequences representing a subset of species belonging to this genus consisting of all available high-quality, near-full-length records available from the RDP. The reference set comprised *Lactobacillus* species that have been shown to be present in the human vagina including *L.crispatus*, *L. iners*, *L. gasseri*, *L. jensenii*, and *L. vaginalis* [17], [18], [23], [33], [34]. One representative sequence from each group of identical sequences were retained for validation. Sequences used for validation also met the same distance criteria as described for the reference set creation to avoid introducing mislabeled records into the analysis. Sequences were trimmed to span the same 16S rRNA region as the query reads. The classification outputs summarizing the placement of the validation set of *Lactobacillus* sequences are provided in the Text S1.

### Community Analysis

**Figure 1:**
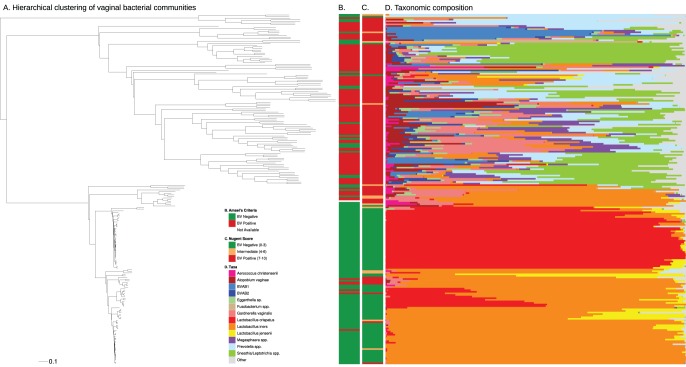
Vaginal bacterial communities in all women. A novel variant of hierarchical clustering was used to generate a clustering tree depicting the bacterial diversity in 220 women with and without BV (A). The scale bar represents KR distance, a generalization of UniFrac (see Methods). The colored bars represent bacterial taxa that were most abundant in each sample. Less abundant taxa are grouped in the “other” category. BV status by Amsel’s criteria (B) and Gram stain (C) is provided for all women in the 2 vertical bars. In the absence of BV, the vaginal microbiota was dominated with *Lactobacillus crispatus* or *Lactobacillus iners*. Women with BV had more diverse bacterial communities.

Distances between samples were calculated using the phylogenetic Kantorovich-Rubinstein (KR) metric, also known as the “earth-mover” distance. As described in [35], the KR metric in this context is a generalization of weighted UniFrac [36], allowing assignments to internal edges of the tree and for mass to be split according to probabilistic assignment. In general terms, this metric considers each sample to be a distribution of one total unit of mass across the tree, where mass is deposited on edges in proportion to the number of reads which map to that edge. The earth-mover distance, then, quantifies the difference between two different distributions of masses by considering the amount of work to move one distribution to the other. For Figure 1, we implemented a novel variant of hierarchical clustering, which we call “squash clustering” [37]. UPGMA or average-linkage clustering is an hierarchical clustering method by which the matrix-updating procedure is done by calculating the distance between clusters A and B as the average of the distances between samples in A and B. Squash clustering is similar but vice versa: the distance between two clusters is defined to be the distance between the averages of the mass distributions. The name comes from the mental image of stacking the mass distributions on top of one another and “squashing” them down so that they have unit total mass. Internal branch lengths are assigned by calculating distances between these “squashed” cluster masses. This meaningful internal branch length calculation implies that the clustering trees may not be ultrametric (or “clocklike”) in contrast to the typical representation of a UPGMA tree. Bootstrap analysis is performed by resampling (with replacement) from the collection of phylogenetic placements for each sample individually.

Edge principal components analysis is an ordination method that takes advantage of the structure of the phylogenetic tree. Edge PCA performs a transform of the placement data using the structure of the phylogenetic tree to get a collection of vectors, one per sample, indexed by the edges of the tree. Classical PCA is then applied to these vectors; the resulting eigenvectors can be visualized on the tree [37]. All of these calculations were performed with our guppy software, available at http://matsen.fhcrc.org/pplacer/.

### Ecological Characterization

Statistics of ecological diversity [38] and richness [39] of reads classified to their most specific rank were calculated separately for each sample, using implementations in the vegan package in R [40] and facilitated by the *microbiome* package.

Unsupervised machine learning approaches were used to summarize relationships between samples and explore taxonomic associations with BV classification. Taxa were trimmed by ranking from most to least abundant (across all samples), and retaining the top 30%; these taxa accounted for >98.5% of the classified reads. Samples were clustered using log-transformed richness estimates (with a constant 0.5 added as continuity correction), a Euclidean distance metric, and complete linkage. Calculations used the R functions *dist* and *hclust*; visualization used the *heatmap* function. Co-occurrence of taxonomic groups across samples was explored by calculating the Pearson correlation between log-transformed richness estimates of all pairs of taxa across samples, and clustering these correlations using *dist* and *hclust* as described above.

### Characterization of Clostridiales Sequences

To identify potentially novel and interesting *Clostridiales* bacteria detected in this study, all pyrosequencing reads classified as belonging to this order were submitted to a phylogenetic-based clustering approach after placement on the reference tree. Reads were clustered into phylogenetic “islands” using `guppy compress̀ with a distance cutoff of 0.02. Islands were considered further only if reads originated from at least two different subjects; remaining islands were labeled according to the classification of constituent reads. For each island, an arbitrarily selected representative sequence was searched against the NCBI nr nucleotide database using BLAST with the filter query ‘all [filter] NOT(environmental samples[filter] OR metagenomes[orgn])’. Islands were represented as individual leaves on the reference tree using the ‘guppy mft’ and ‘guppy tog’ commands (Figure S1).

### Association of Microbiota with Race

Associations between race and BV status were assessed using a general linear model approach, applied to each taxon. The model attempts to accommodate over-representation of zero counts by separately modeling zero and non-zero components [41]. The non-zero component of the model includes dependent variables race, BV status, and their interaction. For pragmatic reasons the zero component is modeled such that all zero counts have the same probability of belonging to the zero component; fits used a negative binomial error model. Our interest is in the interaction between race and BV status, so our test compares the goodness of fit of the full model (main effects and interaction) with a reduced model (main effects alone). Models were fit using the *zeroinfl* function of the *pscl* R package [42], with comparison between the full and reduced model using the *anova* base R function. Reported P-values are Benjamini-Hochberg adjusted to control false discovery rate. Assessment of model significance across each taxon is facilitated by the *microbiome* package.

### Bacterial Associations with Clinical Criteria

Associations between bacterial taxonomic composition and Amsel’s clinical criteria were assessed using penalized linear (‘elasticnet’) models. The approach is to model a sign, e.g., vaginal pH, as a linear function of log-transformed bacterial counts in each sample. The coefficients of the regression model are constrained in overall magnitude, typically forcing some coefficients toward zero. The magnitude of the overall constraint is characterized using cross validation methods [43], with coefficients reported for taxa with non-zero coefficients in the fitted model. Models were fit using the *cv.glmnet* function of the *glmnet* R package [44].

## Results

Of the 220 women studied by molecular evaluation of their microbiota, 98 (43%) had BV by Amsel’s criteria, and 117 (53%) by Gram stain (Table 1). Meta data for individual study participants is provided in Table S3. Only 33% of all study participants (73/220) had a pH of ≤4.5, which is considered “normal” vaginal pH, though 55% of all women (121/220) in the study did not have BV by clinical criteria. Thirty-four percent were Black and 44% were White women. Although study participants were recruited from an STD clinic, there was low prevalence of *Trichomonas vaginalis* infection (7%). Vulvovaginal candidiasis was also rare (8%).

**Table 1:** Study participant characteristics and demographics.

Participant Characteristic	
**Age**	
Age range	18–57 years
Mean	29.2 years
Median	27.3 years
1 **Race**	2 **N (%)**
Black	75 (34%)
White	97 (44%)
Other	48 (24%)
**BV Diagnosis by Amsel’s criteria**	**N (%)**
Negative	121 (55%)
Positive	98 (44.5%)
**Amsel’s criterion – Vaginal discharge**	3 **N (%)**
Normal	97 (44%)
Abnormal	103 (47%)
**Amsel’s criterion - pH**	**N (%)**
4.5 and less	73 (33%)
Greater than 4.5	145 (66%)
**Amsel’s criterion - Clue Cells**	**N (%)**
Absent	95 (43%)
Less than 20% (few)	28 (13%)
More than 20% (many)	79 (36%)
**Amsel’s criterion - Whiff Test**	**N (%)**
Negative	132 (60%)
Positive	87 (40%)
**BV Diagnosis by Nugent Score**	**N (%)**
Negative	103 (47%)
Positive	117 (53%)
**Other infections**	**N (%)**
*Trichomonas*	15 (7%)
Yeast	17 (8%)

1 The race data represents women who report a single race. Data was also collected for other races including Asian, American Indian/Alaskan Native and Native Hawaiian/Pacific Islander but not shown in the table as each group was represented by less than 10% of the study cohort.

2 N indicates number of participants. Not every data element was provided by every subject.

3 Amsel’s criteria not available for one study participant.

We generated a dataset of 425775 sequence reads meeting quality criteria from 220 samples with a median read length of 225 bp and a mean of 1620 sequence reads per study participant (SRA051298). Sequence reads were passed through our taxonomic classification pipeline by placing reads in a phylogenetic tree broadly representing vaginal bacteria. Classification was achieved for 99.1% of sequence reads at species level, 0.8% at genus level, and 0.1% at higher taxonomic levels. The taxonomic assignments of the sequence reads in all women along with BV status are displayed in Table S4.

### Vaginal Bacterial Communities in Women with and without BV

**Figure 2:**
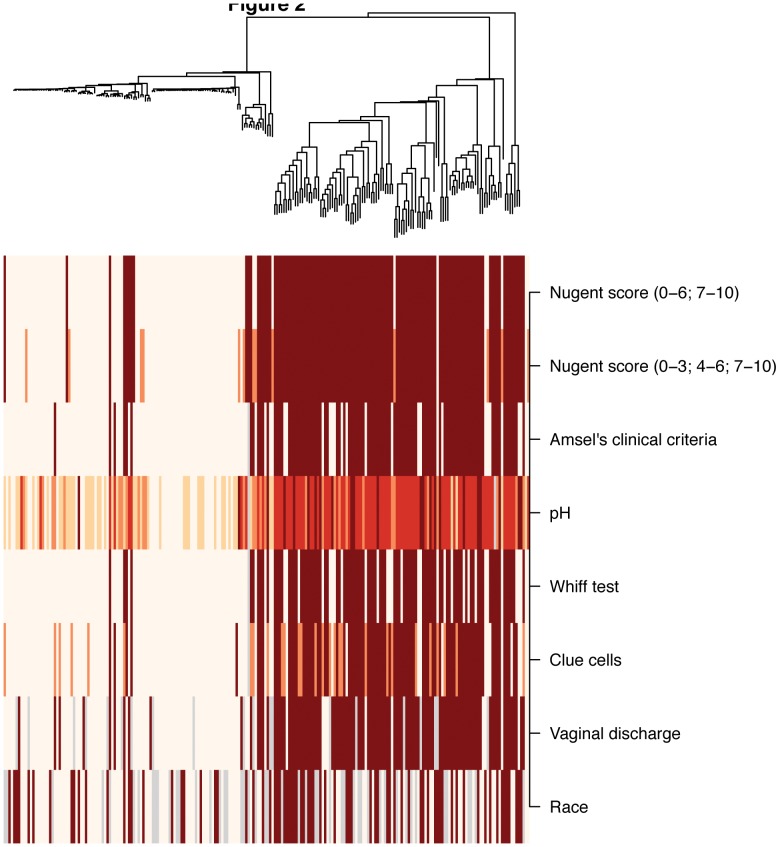
Association of vaginal bacterial communities with BV status, clinical features and race. A dendrogram shows association of meta data with vaginal bacterial communities. Nugent score and Amsel’s clinical criteria for BV are expressed as negative (light) and positive (dark). Nugent scores are presented in 2 formats, one considering BV positive (7–10) vs. BV negative (0–6), and the other separating data on women with intermediate scores (4–6) indicated in orange vs. women without BV (0–3) in white). pH values range from 4 to 6. Numerical values are shaded from light (low) to dark (high). Whiff test is expressed as positive (dark) or negative (light) and abundance of clue cells is expressed as none, <20%, and >20% (light to dark). Vaginal discharge is represented as normal (light) and abnormal (dark). The race band denotes white (light) and black (dark) women. Gray bars denote that data not available or do not fit the criteria outlined. Meta data are also available in Table S3.

Vaginal bacterial community composition superposed on the squash clustering tree showed that women with BV have diverse, heterogeneous communities. In contrast, women without BV are colonized primarily with *Lactobacillus* species (Figure 1). Vaginal bacterial community association with additional metadata is shown in Figure 2. Median levels of species richness and diversity of bacteria were higher in women with BV (Figure S2). Among women with BV, four clusters were dominated by a single taxon, including BVAB1, *Prevotella* spp. and two dominated by *Sneathia sanguinegens/Leptotrichia amnionii* (Figure 1). All other clusters contained multiple bacterial taxa without a dominant bacterium. Although presence/absence data showed that 47 bacterial taxa were significantly associated with BV (asymptotic adjusted p-value <0.05) (Table S5), 95% of the sequence reads from women with BV were accounted for by the top 24 taxa and included bacteria such as BVAB1, BVAB2, *Megasphaera* spp., *L. amnionii*, *S. sanguinegens*, *Gardnerella vaginalis*, *Atopobium vaginae* and others. *G. vaginalis* was present in 97.4% of women with BV accounting for 15% of sequence reads while *Lactobacillus iners* was present in 86.3% of women with BV accounting for 17.9% of reads (Tables S4). Other bacteria that were present in greater than 75% of women and significantly associated with BV included *A. vaginae* (92%), *Eggerthella*-like (85.5%), *L. amnionii* (78%), *Aerococcus christensenii* (79%), *Dialister micraerophilus* (79%) and *Prevotella timonensis* (75%) (Table S5). Ninety-three percent of women without BV (by Gram stain) had vaginal bacterial communities dominated by either *Lactobacillus crispatus* or *L. iners* (Figure 1, table S4). *Lactobacillus jensenii* was the dominant taxon in 2 women (1.9%). Five women without BV (4.9%) had different dominant bacteria including *A. vaginae*, *L. amnionii*, *Prevotella amii*, BVAB1 and *Fusobacterium gonidiaformans*. Of these 5 women, 4 had intermediate Nugent scores. Other abundant lactobacilli in women without BV included *L. jensenii* and *Lactobacillus gasseri*, present in 65% and 34% of women respectively. We also detected *Lactobacillus vaginalis* (27%) and *Lactobacillus coleohominis* (15%) as minor members of the vaginal communities in women without BV.

**Figure 3:**
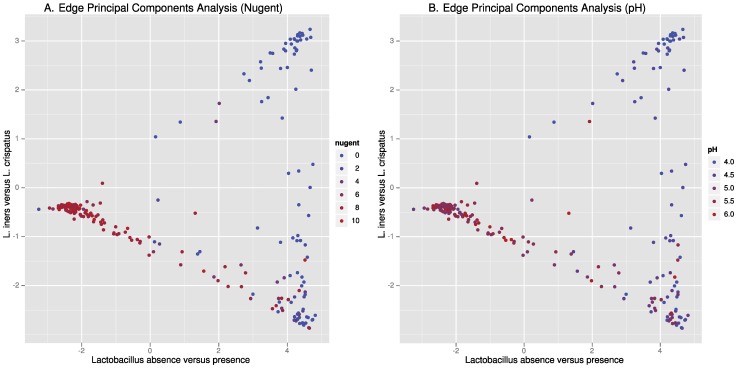
*Lactobacillus* species profiles in relation to BV status (A) and pH (B). Edge principal components analysis, a variant of classical principal components analysis facilitates the labeling of the two components. The algorithm was not informed by the Nugent score or taxonomic classifications and the axes were independently selected by the algorithm. The x-axis represents presence of *Lactobacillus* species and the y-axis represents abundance of the two key *Lactobacillus* species, *Lactobacillus crispatus* and *Lactobacillus iners*. BV status is indicated by Nugent score; 0–6: BV Negative, 7–10: BV positive.

The presence of *Lactobacillus* species is a major portion of the first component distinguishing women with and without BV in the edge-PCA analysis, corroborating the squash clustering (Figure 3). *L. iners* and *L. crispatus*, the dominant lactobacilli in our dataset, contribute to the second principal component. The PCA was not informed of Nugent score or taxonomic classifications. The edge PCA vectors projected on to the phylogenetic tree are provided in Figures S3A and S3B. While women with high levels of *L. crispatus* did not have BV, women with high levels of *L. iners* could be either BV negative or positive. A lack of *Lactobacillus* species is typically associated with having BV (see red data points on left of Figure 3A), and this is concordant with detection of *Lactobacillus* morphotypes on Gram stain to categorize BV status. As levels of *L. iners* decrease (moving from right lower corner of plot to left mid zone), there is a continuum of Nugent scores leading to the BV positive group. This continuum was not observed with *L. crispatus* (Figure 3A). Presence/absence analysis further supported the interpretation that *L. iners* was not associated with absence of BV (Table S5).

### New Bacterial Species Detected in the Vagina

Deep sequencing coupled with species-level classification revealed an abundance of yet-unnamed bacteria, indicating that there is unappreciated species diversity in the human vagina. Among all reads in the *Clostridiales* Order, we detected sequences representing 8 families (Table 2, Figure S1). Using BLAST searches of representative sequences against the nr nucleotide database, we identified fourteen sequence types present in at least two samples having less than 97% sequence identity to named sequences in GenBank. Among these, three species candidates, BVAB-1, BVAB-2 and BVAB-3, have been described previously by our lab [18] (Table S6). The sequence identities for the other 11 sequence groups to the most similar named records ranged from 91.2% to 96.6%.

**Table 2:** Potential novel bacteria identified in the *Clostridiales* Order.

Classified As	1Cluster Number	2Numberof Reads	3Numberof Samples	Closest BLAST Hit	Sequence Identity	Accession Number
Clostridiales	C10	10	5	*Lachnobacterium bovis* strain LRC 5436	91.2	AF298665
Peptoniphilus	C26	3	2	*Peptoniphilus lacrimalis* strain CCUG 31350	92.5	NR_041938
Lachnospiraceae	C12	115	12	*Syntrophococcus sucromutans* strain S195	93.3	NR_036869
Parvimonas micra	C35	228	7	*Parvimonas micra* strain 3119B	93.5	NR_036934
Clostridiales/Clostridium colicanis	C3	170	15	*Eubacterium tarantellae* type strain DSM3997T	93.6	FR733677
Ruminococcus obeum	C7	6	2	*Eubacterium cf. saburreum* strain C27KA	93.7	AF287777
Peptoniphilus	C24	12	10	*Peptoniphilus asaccharolyticus* strain JCM 1765	93.8	AB640690
Lachnospiraceae	C8	13	4	*Syntrophococcus sucromutans* strain S195	94.3	NR_036869
Lachnospiraceae	C4	20	4	*Roseburia intestinalis* strain JCM 17583	95.6	AB661435
Peptoniphilus	C25	63	19	*Peptoniphilus methioninivorax* strain NRRL B-23883	96.1	GU440754
Clostridiales	C2	23	7	*Eubacterium tarantellae* type strain DSM3997T	96.6	FR733677

1 Cluster number is a unique identifier of each cluster and is shown in the tree representing all clusters of sequence reads in the *Clostridiales* Order (Supplementary Figure S1).

2 Number of Reads refers to the total number of reads in the dataset.

3 Number of Samples refers to the number of samples in which the sequence type was detected.

### Sub-types of Bacterial Communities in BV

**Figure 4:**
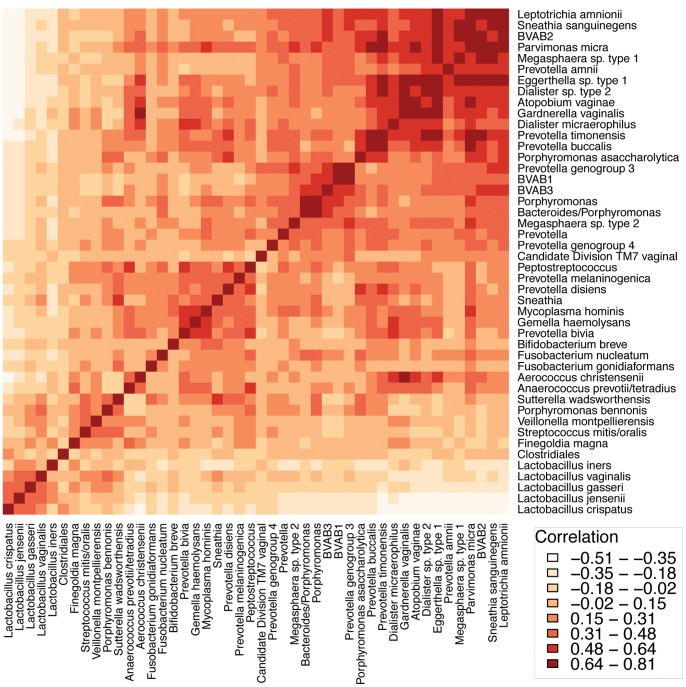
Co-occurrence analysis of bacterial taxa. Hierarchically clustered Pearson correlation coefficients between abundant bacterial taxa are displayed. Correlation values range from −0.51 (light) to 0.81 (dark). Diagonal elements have correlation of 1. Several sub-groups of bacteria associated with BV show strong positive associations. The coefficients are available in Table S7.

Co-occurrence of bacterial taxa was investigated for the most abundant taxa by calculating Pearson correlation coefficients (Figure 4, Table S7). The lactobacilli were strongly correlated with each other, as were the BV-associated anaerobes. We observed strong negative correlations between most lactobacilli and the bacteria associated with BV. Among the BV-associated bacteria, there were several sub-groups with strong positive correlations between bacteria suggesting metabolic or other dependencies; bacteria that are negatively correlated may compete for similar nutrients or change the environment in ways that inhibits growth of each other. In our study, the largest sub-group of positively associated bacteria comprised BVAB2, *Megasphaera* sp. type 1, *L. amnionii, S. sanguinegens, G. vaginalis*, *A. vaginae*, *Eggerthella*, *Parvimonas micra*, *Prevotella buccalis*, *Prevotella amnii, Dialister micaerophilus* and *Dialister* sp. 2. Another sub-group consisted of BVAB1, BVAB3 and *Prevotella* genogroup 3. *G. vaginalis* had the strongest positive correlation with *Eggerthella*, *Dialister* sp. type 2, *A. vaginae* and *Aerococcus christensenii*. *L. crispatus* had strong positive correlations with *L. jensenii* and *L. gasseri*, but was negatively correlated with *L. iners*.

### Vaginal Bacterial Communities and Race

**Figure 5:**
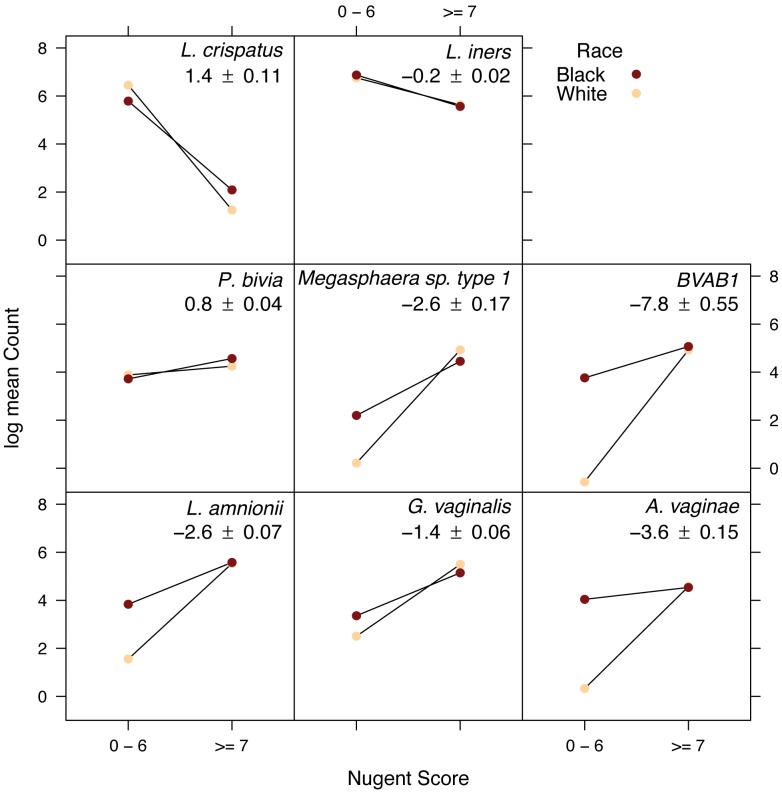
Association of race with vaginal microbiota. A general linear model (see Methods for additional detail) was used to describe interactions between race and BV status as determinants for bacterial prevalence. On the x-axis, BV was assessed by Gram stain: 0–6, BV negative; 7–10, BV positive. On the y-axis counts were sequence reads in each group; values summarized in the figure are log-transformed average counts in each group. Values below each taxonomic identifier are the estimate of the strength of the interaction +/− one standard error; large positive values indicate a more positive (less negative) slope amongst Black women than White women. Eight of 28 bacterial taxa showing greatest interaction with race are displayed.

Black women have a consistently higher prevalence of BV compared to other racial groups [45]. Twenty-eight bacterial taxa exhibited changes in BV-associated prevalence that depend on race (Table S8; p<0.05, adjusted for multiple comparisons). Highly significant interactions including *L. amnionii*, *G. vaginalis*, *A. vaginae*, *Megasphaera* sp. type 1 and BVAB1 are displayed in Figure 5. Race and BV status interact to influence the microbiota.

### Association of Bacterial Taxa with Clinical Features

**Figure 6:**
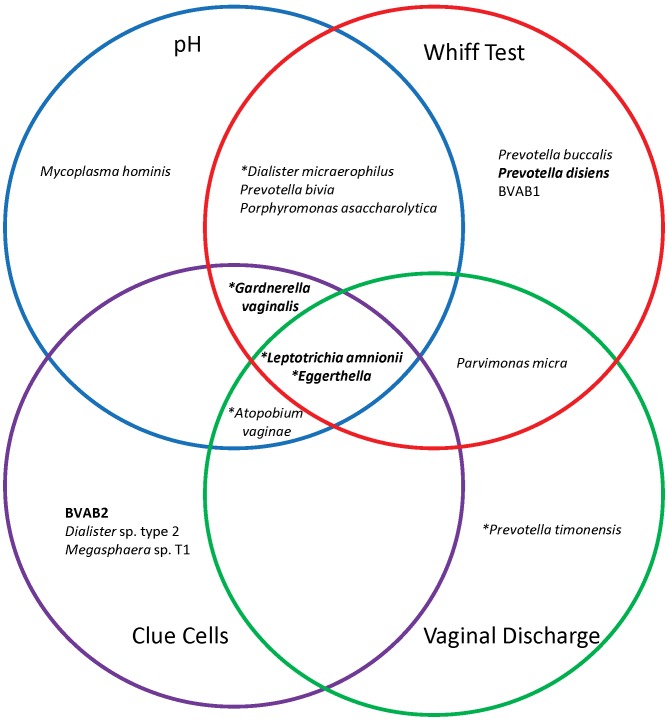
Bacterial taxa associated with Amsel’s clinical characterisitics. A model representing all bacterial taxa associated with the four clinical variables used to diagnose BV. *Leptotrichia amnionii* and *Eggerthella* sp. were found to be associated with each of the four clinical criteria. Stars denote bacteria that were present in greater than 75% of women with BV. Taxa in bold denote those showing associations with Amsel’s criteria as a composite unit.

We examined associations of Amsel’s criteria with bacterial taxa using penalized linear models (Figure 6, Table S9). *Eggerthella* and *L. amnionii* were the only BV-associated bacteria that were positively associated with all four clinical criteria. *L. crispatus* was strongly correlated with the absence of BV and was the only *Lactobacillus* species associated with low pH, negative whiff test, absence of clue cells and normal vaginal discharge (Table S9). *G. vaginalis* and *A. vaginae* were each associated with 3 criteria. *G. vaginalis* was not associated with abnormal vaginal discharge, while *A. vaginae* was not associated with amine odor.

We used edge PCA to determine the effects of the two dominant lactobacilli on pH. Women with high levels of *L. crispatus* have low pH (Figure 3B). In contrast, women with high *L. iners* levels can have either low or high pH.

## Discussion

In many clinics, women are diagnosed with BV according to the Amsel criteria when ≥3 clinical criteria are present, including vaginal pH >4.5, amine odor on addition of KOH to vaginal fluid, thin homogeneous discharge, and presence of shed vaginal epithelial cells coated with bacteria referred to as clue cells [8]. Little is known about the relationship of individual bacteria with the clinical findings and symptoms of BV. We hypothesized that different bacteria or groups of bacteria are associated with each of the clinical criteria for BV, partly explaining the complexity of this polymicrobial condition. Only two bacteria, *Leptotrichia amnionii* and *Eggerthella* sp., were associated with each of the 4 clinical criteria for BV, though many taxa were significantly correlated with BV overall. *Gardnerella vaginalis* and *Atopobium vaginae* (among other bacteria) were associated with the presence of clue cells, and interestingly, both bacteria have been detected in BV biofilms [46], [47], [48]. The fishy odor in vaginal discharge is attributed to polyamines such as putrescine, cadaverine, and trimethylamine [49], [50]. Several bacteria including *Prevotella* spp., BVAB1 and *Dialister micraerophilus* were associated with a positive whiff test (amine odor). These polyamines are thought to arise from bacterial metabolism, and these 3 bacteria are candidates for their production. Future bacterial cell culture and co-culture experiments can help shed light on the metabolic capabilities of individual BV-associated bacteria which will aid in shaping our strategies for prevention of BV. Another study of the vaginal microbiota showed that amine odor was associated with *Atopobium vaginae* and *Veillonellaceae* [12]. Only *Lactobacillus crispatus* was negatively associated with all four clinical criteria and women with high levels of *L. crispatus* had low vaginal pH; this is in agreement with results reported by another group [12]. It is widely described in the literature that *L. crispatus* is important for the maintenance of health in the vagina [51], [52], [53], yet this bacterium is not universally present in women without BV. Women who don’t have BV often have vaginal microbiotas dominated by *Lactobacillus iners,* yet *L. iners* is also present in high numbers in women with BV. Indeed, edge-PCA analysis suggested that the vaginal fluid in women with high levels of *L. iners* can have either high or low pH, and many with high pH have BV. It is possible that *L. iners* facilitates transitions between BV and non-BV states, and that the development of BV requires the presence of other BV-associated bacteria. This hypothesis is consistent with our longitudinal data showing that *L. iners* is frequently the dominant vaginal bacterium after treatment for BV [46]. In our analysis associating bacterial taxa with clinical features, *L. iners* was not associated with any of the Amsel’s clinical criteria for BV, and in fact, was also not associated with the absence of clinical characteristics.

Women with BV had heterogeneous bacterial communities with increased species richness and species diversity. No bacterium was present in all women with BV, although *G. vaginalis* was present in most (98%). Other bacteria present in significant numbers in women with BV included *A. vaginae* (92%), *L. iners* (86%) and *Eggerthella* species (85%). It is possible that the bacterial heterogeneity in BV is due to functional redundancy between bacterial species, such that the presence of either species “A” or “B” in the community fills some functional role, but not presence of both. Bacterial heterogeneity is common in other body sites including the gut, skin and oral cavity [54], [55], [56], [57], [58] and metagenomic studies indicate that these microbiota can maintain remarkable stability in function while undergoing major shifts in species composition [59].

The healthy human vagina is dominated by *Lactobacillus* species [18], [33], [60] despite the presence of complex communities of bacteria in close proximity on the skin and anus. In contrast, there is no single dominant bacterium in the normal human intestine. One goal of the Human Microbiome Project is to determine if there is a core microbiome, or group of microbes (and their genes) that are shared by all humans. For the human vagina, there does not appear to be a true core of bacterial species that are universally shared, though distinct community profiles are evident. The nature of these community profiles is a matter of ongoing debate. In this study, we confirm that women without BV have vaginal microbiotas dominated with *Lactobacillus* species, notably either *L. crispatus* or *L. iners*. One research team proposed the existence of five bacterial community groups in asymptomatic women, of which four are dominated with a different *Lactobacillus* species including *L. iners*, *L. crispatus*, *L. jensenii* or *L. gasseri* [17], [61], [62]. The fifth group comprised diverse bacteria and lactobacilli without a dominant species and resembles BV-associated bacterial communities, but these studies did not assess BV status using Amsel’s criteria, and many of these subjects had BV by Nugent score. *L. iners* was found to be the predominant *Lactobacillus* species in a group of both HIV^+^ and HIV^−^ women [34]. Hummelen *et al*. proposed that *G. vaginalis* and *L. iners* were present in all women regardless of BV status and hence constitute core members of the vaginal microbiota [12]. We have previously used highly sensitive species-specific PCR to show that *G. vaginalis* and *L. iners* are not universally present in the human vagina [63]. In the present study, we detected *L. iners* in 90% of women and it was the only *Lactobacillus* species that did not correlate strongly with absence of BV (p = 0.75). The second most prevalent bacterium in this study was *G. vaginalis* in 70% of all women.

An important consideration when comparing studies in the literature is the use of primers targeting different hypervariable regions of the 16S rRNA gene. We targeted the V3/V4 region for broad-range PCR and sequenced the V4 region. The V4 hypervariable region is reported to give the lowest error rates when assigning taxonomy [64], [65] and is also suitable for community clustering [66]. Sequences spanning the V4 region tend to generate species richness estimates concordant with those obtained from longer fragments, and hence V4 is recommended as a suitable region for pyrosequencing studies [65]. Regardless of which region of the 16S rRNA gene is used for sequence analysis, efforts to define either a core microbiome or core of distinct community profiles will depend on robust phylogenetic resolution, as demonstrated here. The selection of broad range 16S rRNA gene PCR primers can have a profound impact on the types of bacteria detected. Primer bias can lead to over- or under-representation of specific taxa [46], [67], [68], [69], resulting in different biological conclusions. For example, the commonly used 27F primer is known to have mismatches with the *G. vaginalis* 16S rRNA gene sequence, a key bacterium in the vaginal niche [67], potentially leading to lower representation of these sequences in some studies [17], [61], [62]. Primers targeting the V6 region are biased against *Sneathia*, *Leptotrichia*, *Ureaplasma* and *Mycoplasma* [12], bacteria often detected in the vagina. The V3/V4 region is currently used for the NIH Human Microbiome Project.

Microbial community composition is often driven by bacterial interactions such as antagonistic and synergistic relationships among individual bacterial species. Indeed, assessing the composition of the bacterial community and factors that shape the assembly of the community members is a critical step for understanding ecosystem dynamics and function. Several sub-groups of BV-associated bacteria were strongly correlated with each other suggesting metabolic co-dependencies between these bacteria. For example, BVAB1, which is yet to be cultivated, is strongly associated with BVAB3 and *Prevotella* genogroup 3 indicating that BVAB1 may require particular metabolites that are produced by these two taxa to facilitate growth, or vice versa. Strong negative correlations between most lactobacilli and bacteria associated with BV indicate an antagonistic relationship between these groups, a phenomenon that is well described [70], [71], [72]. These analyses serve as a foundation for further investigation of microbe-microbe interactions in the human vagina, and generate new hypotheses about ways to cultivate fastidious bacteria based on co-cultivation strategies.

Black women have a higher prevalence of BV than White women [73]. We sought to determine if the vaginal bacterial biota in Black women is different from White women when accounting for BV status. In our analysis, 28 bacterial taxa were significantly associated with race, including *Leptotrichia amnionii*, *Atopobium vaginae* and BVAB1. These bacteria were more abundant in Black women than White women without BV. More Black women had vaginal microbiotas dominated by *L. iners*, whereas more White women had microbiotas dominated by *L. crispatus*. It is unclear if these different *Lactobacillus* species profiles in women without BV lead to differences in risk for BV. Ravel *et al*. also observed differences in the vaginal microbiota associated with race. Asymptomatic Black and Hispanic women in North America were more likely to have vaginal bacterial communities comprising diverse bacteria that mirrored bacterial communities typically seen with BV (community group 4) [17]. However, exams to document absence of vaginal discharge were not reported, Amsel’s clinical criteria for BV were not assessed, and many of these women had BV by Gram stain of vaginal fluid. Nevertheless, we concur with Ravel and colleagues that there appear to be differences in vaginal bacterial biota associated with race.

Different bacterial species can have markedly distinct pathogenic or functional potential. Therefore, it is critical to identify bacteria to the species level in order to appreciate their impact on human biology. Most high-throughput studies of the vaginal microbiota have identified bacterial taxa to the genus, family, or order level, but fail to take the additional step of distinguishing between bacterial species [12], [13], [17], [74]. One goal of this study was to describe vaginal bacterial communities in women with BV and compare them with communities in women without BV using high-throughput sequencing and bioinformatics tools that provide species level resolution. For this purpose, we developed a classification approach that uses a reference tree with representative 16S rRNA gene sequences of vaginal origin. The iterative nature of the construction of this reference tree facilitated species level identification of greater than 99% of sequence reads. In contrast, a deep sequencing study of the vaginal microbiota using a different bioinformatics approach and targeting the *cpn60* gene classified only 65% of sequence reads to species level [14]. An important feature of our classifier is the accurate identification of uncultivated yet key BV-associated bacteria such as BVAB1, BVAB2, and *Megasphaera* spp. [18], [75] which are not differentiated using existing classification pipelines such as Greengenes [16] or RDP [15]. This feature of the classifier is essential in the accurate identification of the majority of query sequences in this study. Moreover, we detected 11 novel bacteria within the *Clostridiales* Order that have less that 97% sequence identity with cultivated isolates in GenBank. The potential importance and contribution of rare species of the microbiota is currently under-appreciated mostly due to challenges in reliably describing the rare members of the vaginal microbiota using existing classifiers.

In conclusion, women with BV have complex vaginal bacterial communities with increased species richness and diversity compared to women without BV. Although women with BV have heterogeneous vaginal bacterial populations, there are some clear sub-groups of bacterial communities, such as one dominated by BVAB-1 and others dominated by *Leptotrichia* or *Sneathia* species. Co-occurrence analysis suggests that there are strong positive and negative interactions among some vaginal bacterial species that may drive these community profiles. Women without BV had vaginal bacterial communities dominated by either *L. crispatus* or *L. iners*. Black women without BV have higher abundances of several BV-associated bacterial species that may contribute to increased risk for BV. Different bacterial species appear to impact different elements of the clinical criteria used to diagnose BV. Lastly, we have developed a suite of bioinformatics tools that facilitate species-level microbial classification. These tools can be readily customized to classify high-throughput sequence reads from other human body sites or ecosystems.

## Supporting Information

Figure S1 **Clusters of sequence reads in the** ***Clostridiales*** **Order.** All pyrosequencing reads classified as belonging to the *Clostridiales* Order were submitted to a phylogenetic approach after placement on the reference tree. A cluster number is indicated after each taxon name. Clusters were labeled only if the sequence reads originated from at least two different subjects. The number of reads in a cluster present in the entire data set is also shown. Symbols denote the family level classification for each taxon.
(PDF)

Figure S2 **Descriptive Statistics.** Chao 1 plots comparing species richness and Shannon plots comparing species diversity. Chao Plot 1 (Nugent Score; 0–6 and 7–10): BV is characterized by greater species richness. Chao Plot 2 (Nugent Score; 0–3, 4–6 and 7–10): The median numbers of taxa are similar between those who have Intermediate flora and frank BV. Chao Plot 3 (Amsel’s clinical criteria): Women who are diagnosed for BV by Amsel’s clinical criteria have greater numbers of taxa than those who don’t have BV. Chao Plot 4 (Amsel’s criteria – pH): Higher pH is typically associated with increased species richness. Chao Plot 5 (Amsel’s criteria – Whiff test): Vaginal samples that have an amine odor upon addition of potassium hydroxide are characterized by increased species richness. Chao Plot 6 (Amsel’s criteria – Clue cells): Vaginal samples that have <20% clue cells have similar numbers of taxa when compared to those who have >20% clue cells. Chao Plot 7 (Amsel’s criteria – Vaginal discharge): Women with abnormal vaginal discharge are typically colonized with greater numbers of taxa than those with normal vaginal discharge. Shannon Plot 1 (Nugent Score; 0–6 and 7–10): BV is characterized by increased species diversity. Shannon Plot 2 (Nugent Score; 0–3, 4–6 and 7–10): There is greater species diversity in those who have Intermediate flora (4–6) and frank BV (7–10) when compared with women without BV (0–3). Shannon Plot 3 (Amsel’s clinical criteria): Women who are diagnosed for BV by Amsel’s clinical criteria have greater species diversity than those who don’t have BV. Shannon Plot 4 (Amsel’s criteria – pH): Higher pH is typically associated with increased species diversity. All women with a pH of 4.5 and less were BV negative. Shannon Plot 5 (Amsel’s criteria – Whiff test): Vaginal samples that have an amine odor upon addition of potassium hydroxide are characterized by increased species diversity. Shannon Plot 6 (Amsel’s criteria – Clue cells): Vaginal samples that have clue cells show increased species diversity. Samples with <20% clue cells have similar diversity indices when compared to those who have >20% clue cells. Shannon Plot 7 (Amsel’s criteria – Vaginal discharge): The species diversity is greater in the vaginal microbiotas of women with abnormal vaginal discharge.
(PDF)

Figure S3 **Edge principal component vectors projected on to the phylogenetic tree.** These vectors are indexed by edges of the reference tree, and displayed as colored and thickened edges. The thickness of an edge is proportional to its weight in the principal component vector; positive coefficients marked with orange and negative are marked in green. The first principal component (S3A), with 59% of the variance, has all positive coefficients on edges leading to the *Lactobacillus* clade. The second principal component (S3B), with 17% of the variance, gives a positive coefficient to the *Lactobacillus crispatus* clade and a negative coefficient to the *Lactobacillus iners* clade. These tree diagrams justify the axis labels given to Figure 3 in the main text.
(PDF)

Table S1 **List of pyrosequencing primers.**
(XLSX)

Table S2 **Tally of sequences representing each taxon in the vaginal reference package.**
(XLSX)

Table S3 **Meta data for individual study participants.**
(XLSX)

Table S4 **The taxonomic output of sequence reads in all women along with BV status.**
(XLSX)

Table S5 **Presence/absence patterns of bacterial taxa associated with BV.**
(XLSX)

Table S6 **Bacterial clusters in the** ***Clostridiales*** **Order.**
(XLSX)

Table S7 **Pearson correlation coefficients between the most abundant taxa.**
(XLSX)

Table S8 **Vaginal microbiota and race associations.**
(XLSX)

Table S9 **Association of bacteria with Amsel’s clinical criteria.**
(XLSX)

Text S1 **Classifier Validation.**
(PDF)

Reference Package S1 **(vaginal-0.9.2-raxml.refpkg) Contains files required for classification of vaginal reference sequences in the V3/V4 region of the 16S rRNA gene.**
(TGZ)

## References

[1] Hillier SL, Krohn MA, Cassen E, Easterling TR, Rabe LK (1995). The role of bacterial vaginosis and vaginal bacteria in amniotic-fluid infection in women in preterm labor with intact fetal membranes.. Clin Infect Dis.

[2] Krohn MA, Thwin SS, Rabe LK, Brown Z, Hillier SL (1997). Vaginal colonization by *Escherichia coli* as a risk factor for very low birth weight delivery and other perinatal complications.. J Infect Dis.

[3] Regan JA, Klebanoff MA, Nugent RP, Eschenbach DA, Blackwelder WC (1996). Colonization with group B streptococci in pregnancy and adverse outcome. VIP Study Group.. Am J Obstet Gynecol.

[4] Koumans EH, Sternberg M, Bruce C, McQuillan G, Kendrick J (2007). The prevalence of bacterial vaginosis in the United States, 2001–2004; associations with symptoms, sexual behaviors, and reproductive health.. Sex Transm Dis.

[5] Haggerty CL, Hillier SL, Bass DC, Ness RB (2004). Bacterial vaginosis and anaerobic bacteria are associated with endometritis.. Clin Infect Dis.

[6] Hillier SL, Nugent RP, Eschenbach DA, Krohn MA, Gibbs RS (1995). Association between bacterial vaginosis and preterm delivery of a low-birth-weight infant.. N Engl J Med.

[7] Taha TE, Hoover DR, Dallabetta GA, Kumwenda NI, Mtimavalye LA (1998). Bacterial vaginosis and disturbances of vaginal flora: association with increased acquisition of HIV.. AIDS.

[8] Amsel R, Totten PA, Spiegel CA, Chen KC, Eschenbach D (1983). Nonspecific vaginitis: diagnostic criteria and microbial and epidemiologic associations.. Am J Med.

[9] Nugent RP, Krohn MA, Hillier SL (1991). Reliability of diagnosing bacterial vaginosis is improved by a standardized method of Gram stain interpretation.. J Clin Microbiol.

[10] Hillier S, Marrazzo JM, Holmes KK, Holmes KK, Sparling P-A (2008). Bacterial vaginosis..

[11] Hay PE (1998). Therapy of bacterial vaginosis.. J Antimicrob Chemother.

[12] Hummelen R, Fernandes AD, Macklaim JM, Dickson RJ, Changalucha J (2010). Deep sequencing of the vaginal microbiota of women with HIV.. PLoS One 5: Article No.

[13] Ling Z, Kong J, Liu F, Zhu H, Chen X (2010). Molecular analysis of the diversity of vaginal microbiota associated with bacterial vaginosis.. BMC Genomics.

[14] Schellenberg JJ, Links MG, Hill JE, Dumonceaux TJ, Kimani J (2011). Molecular definition of vaginal microbiota in East African commercial sex workers.. Appl Environ Microbiol.

[15] Cole JR, Wang Q, Cardenas E, Fish J, Chai B (2009). The Ribosomal Database Project: improved alignments and new tools for rRNA analysis.. Nucleic Acids Res.

[16] DeSantis TZ, Hugenholtz P, Larsen N, Rojas M, Brodie EL (2006). Greengenes, a chimera-checked 16S rRNA gene database and workbench compatible with ARB.. Appl Environ Microbiol.

[17] Ravel J, Gajer P, Abdo Z, Schneider GM, Koenig SS (2010). Microbes and Health Sackler Colloquium: Vaginal microbiome of reproductive-age women.. Proc Natl Acad Sci U S A.

[18] Fredricks DN, Fiedler TL, Marrazzo JM (2005). Molecular identification of bacteria associated with bacterial vaginosis.. N Engl J Med.

[19] Fredricks DN, Fiedler TL, Thomas KK, Mitchell CM, Marrazzo JM (2009). Changes in vaginal bacterial concentrations with intravaginal metronidazole therapy for bacterial vaginosis as assessed by quantitative PCR.. J Clin Microbiol.

[20] Neefs JM, Van de Peer Y, De Rijk P, Chapelle S, De Wachter R (1993). Compilation of small ribosomal subunit RNA structures.. Nucleic Acids Res.

[21] Daims H, Bruhl A, Amann R, Schleifer KH, Wagner M (1999). The domain-specific probe EUB338 is insufficient for the detection of all Bacteria: development and evaluation of a more comprehensive probe set.. Syst Appl Microbiol.

[22] Hamady M, Walker JJ, Harris JK, Gold NJ, Knight R (2008). Error-correcting barcoded primers for pyrosequencing hundreds of samples in multiplex.. Nat Methods.

[23] Oakley BB, Fiedler TL, Marrazzo JM, Fredricks DN (2008). The diversity of human vaginal bacterial communities and their association with clinically-defined bacterial vaginosis.. Appl Environ Microbiol.

[24] El Aila NA, Tency I, Claeys G, Verstraelen H, Saerens B (2009). Identification and genotyping of bacteria from paired vaginal and rectal samples from pregnant women indicates similarity between vaginal and rectal microflora.. BMC Infect Dis.

[25] Verhelst R, Verstraelen H, Claeys G, Verschraegen G, Delanghe J (2004). Cloning of 16S rRNA genes amplified from normal and disturbed vaginal microflora suggests a strong association between *Atopobium vaginae*, *Gardnerella vaginalis* and bacterial vaginosis.. BMC Microbiol.

[26] Clarridge JE 3rd (2004). Impact of 16S rRNA gene sequence analysis for identification of bacteria on clinical microbiology and infectious diseases.. Clin Microbiol Rev.

[27] Edgar RC (2010). Search and clustering orders of magnitude faster than BLAST.. Bioinformatics (Oxford).

[28] Nawrocki EP, Eddy SR (2007). Query-dependent banding (QDB) for faster RNA similarity searches.. PLoS Comput Biol.

[29] Nawrocki EP, Kolbe DL, Eddy SR (2009). Infernal 1.0: inference of RNA alignments.. Bioinformatics (Oxford).

[30] Stamatakis A (2006). RAxML-VI-HPC: maximum likelihood-based phylogenetic analyses with thousands of taxa and mixed models.. Bioinformatics (Oxford, England).

[31] Stamatakis A, Ludwig T, Meier H (2005). RAxML-III: a fast program for maximum likelihood-based inference of large phylogenetic trees.. Bioinformatics (Oxford, England).

[32] Matsen FA, Kodner RB, Armbrust EV (2010). *pplacer*: linear time maximum-likelihood and Bayesian phylogenetic placement of sequences onto a fixed reference tree.. BMC Bioinformatics.

[33] Hyman RW, Fukushima M, Diamond L, Kumm J, Giudice LC (2005). Microbes on the human vaginal epithelium.. Proc Natl Acad Sci USA.

[34] Spear GT, Gilbert D, Landay AL, Zariffard R, French AL (2011). Pyrosequencing of the genital microbiotas of HIV-seropositive and -seronegative women reveals *Lactobacillus iners* as the predominant *Lactobacillus* Species.. Appl Environ Microbiol.

[35] Evans SN, Matsen FA (2012). The phylogenetic Kantorovich-Rubinstein metric for environmental sequence samples. J ROyal Stat Soc (B).. In press.

[36] Lozupone CA, Hamady M, Kelley ST, Knight R (2007). Quantitative and qualitative beta diversity measures lead to different insights into factors that structure microbial communities.. Appl Environ Microbiol.

[37] Matsen FA, Evans SN (2011). Edge principal components and squash clustering: using the special structure of phylogenetic placement data for sample comparison.. http://arXiv.org/abs/1107.5095v1.

[38] Shannon CE (1948). A mathematical theory of communication.. Bell System Technical Journal.

[39] Chao A (1987). Estimating the population size for capture-recapture data with unequal catchability.. Biometrics.

[40] Oksanen J, Guillaume Blanchet F, Kindt R, Legendre P, O’Hara RB (2010). vegan: Community Ecology Package. R package version 1.17–4.. http://CRANR-project.org/package=vegan.

[41] Zeileis A, Kleiber C, Jackman S (2008). Regression models for count data in R. J Stat Soft.

[42] Jackman S (2011). pscl: Classes and methods for R developed in the Political Science Computational Laboratory, Stanford University, Stanford, CA. R package version 1041.. http://pscl.stanford.edu/.

[43] Hastie T, Tibshriani R, Friedman J, editors (2009). The elements of statisitcal learning. New York: Springer.. 754 p.

[44] Friedman J, Hastie T, Tibshirani R (2010). Regularization paths for generalized linear models via coordinate descent.. J Stat Soft.

[45] Peipert JF, Lapane KL, Allsworth JE, Redding CA, Blume JD (2008). Bacterial vaginosis, race, and sexually transmitted infections: Does race modify the association?. Sex Trans Dis.

[46] Srinivasan S, Fredricks DN (2008). The human vaginal bacterial biota and bacterial vaginosis.. Interdiscip Perspect Infect Dis.

[47] Swidsinski A, Mendling W, Loening-Baucke V, Ladhoff A, Swidsinski S (2005). Adherent biofilms in bacterial vaginosis.. Obstet Gynecol.

[48] Swidsinski A, Mendling W, Loening-Baucke V, Swidsinski S, Dorffel Y (2008). An adherent *Gardnerella vaginalis* biofilm persists on the vaginal epithelium after standard therapy with oral metronidazole.. Am J Obstet Gynecol 198: 97 e91–96.

[49] Hillier SL (1993). Diagnostic microbiology of bacterial vaginosis.. Am J Obstet Gynecol.

[50] Wolrath H, Forsum U, Larsson PG, Boren H (2001). Analysis of bacterial vaginosis-related amines in vaginal fluid by gas chromatography and mass spectrometry.. J Clin Microbiol.

[51] Antonio MA, Hawes SE, Hillier SL (1999). The identification of vaginal *Lactobacillus* species and the demographic and microbiologic characteristics of women colonized by these species.. J Infect Dis.

[52] Marrazzo JM, Thomas KK, Fiedler TL, Ringwood K, Fredricks DN (2010). Risks for acquisition of bacterial vaginosis among women who report sex with women: A cohort study.. PLoS One 5: Article No.

[53] Zozaya-Hinchliffe M, Lillis R, Martin DH, Ferris MJ (2010). Quantitative PCR assessments of bacterial species in women with and without bacterial vaginosis.. J Clin Microbiol.

[54] Bik EM, Long CD, Armitage GC, Loomer P, Emerson J (2010). Bacterial diversity in the oral cavity of 10 healthy individuals.. The ISME J.

[55] Costello EK, Lauber CL, Hamady M, Fierer N, Gordon JI (2009). Bacterial community variation in human body habitats across space and time.. Science (New York, N Y ).

[56] Grice EA, Kong HH, Conlan S, Deming CB, Davis J (2009). Topographical and temporal diversity of the human skin microbiome.. Science (New York, N Y ).

[57] Nasidze I, Quinque D, Li J, Li M, Tang K (2009). Comparative analysis of human saliva microbiome diversity by barcoded pyrosequencing and cloning approaches.. Anal Biochem.

[58] Turnbaugh PJ, Hamady M, Yatsunenko T, Cantarel BL, Duncan A (2009). A core gut microbiome in obese and lean twins.. Nature.

[59] Proctor LM (2011). The Human Microbiome Project in 2011 and Beyond.. Cell Host Microbe.

[60] Vasquez A, Jakobsson T, Ahrne S, Forsum U, Molin G (2002). Vaginal *Lactobacillus* flora of healthy Swedish women.. J Clin Microbiol.

[61] Zhou X, Brown CJ, Abdo Z, Davis CC, Hansmann MA (2007). Differences in the composition of vaginal microbial communities found in healthy Caucasian and black women.. ISME J.

[62] Zhou X, Hansmann MA, Davis CC, Suzuki H, Brown CJ (2010). The vaginal bacterial communities of Japanese women resemble those of women in other racial groups.. FEMS Immunol Med Microbiol.

[63] Fredricks DN, Fiedler TL, Thomas KK, Oakley BB, Marrazzo JM (2007). Targeted PCR for detection of vaginal bacteria associated with bacterial vaginosis.. J Clin Microbiol.

[64] Wang Q, Garrity GM, Tiedje JM, Cole JR (2007). Naive Bayesian classifier for rapid assignment of rRNA sequences into the new bacterial taxonomy.. Appl Environ Microbiol.

[65] Youssef N, Sheik CS, Krumholz LR, Najar FZ, Roe BA (2009). Comparison of species richness estimates obtained using nearly complete fragments and simulated pyrosequencing-generated fragments in 16S rRNA gene-based environmental surveys.. Appl Environ Microbiol.

[66] Liu Z, Lozupone C, Hamady M, Bushman FD, Knight R (2007). Short pyrosequencing reads suffice for accurate microbial community analysis.. Nucleic Acids Res.

[67] Frank JA, Reich CI, Sharma S, Weisbaum JS, Wilson BA (2008). Critical evaluation of two primers commonly used for amplification of bacterial 16S rRNA genes.. Appl Environ Microbiol.

[68] Kanagawa T (2003). Bias and artifacts in multitemplate polymerase chain reactions (PCR).. J Biosci Bioeng.

[69] von Wintzingerode F, Gobel UB, Stackebrandt E (1997). Determination of microbial diversity in environmental samples: pitfalls of PCR-based rRNA analysis.. FEMS Microbiol Rev.

[70] Aroutcheva A, Gariti D, Simon M, Shott S, Faro J (2001). Defense factors of vaginal lactobacilli.. Am J Obstet Gynecol.

[71] Atassi F, Servin AL (2010). Individual and co-operative roles of lactic acid and hydrogen peroxide in the killing activity of enteric strain *Lactobacillus johnsonii* NCC933 and vaginal strain *Lactobacillus gasseri* KS120.1 against enteric, uropathogenic and vaginosis-associated pathogens.. FEMS Microbiol Lett.

[72] Matu MN, Orinda GO, Njagi ENM, Cohen CR, Bukusi EA (2010). In vitro inhibitory activity of human vaginal lactobacilli against pathogenic bacteria associated with bacterial vaginosis in Kenyan women.. Anaerobe.

[73] Allsworth JE, Peipert JF (2007). Prevalence of bacterial vaginosis: 2001–2004 National Health and Nutrition Examination Survey data.. Obstet Gynecol.

[74] Price LB, Liu CM, Johnson KE, Aziz M, Lau MK (2010). The effects of circumcision on the penis microbiome.. PloS One.

[75] Zozaya-Hinchliffe M, Martin DH, Ferris MJ (2008). Prevalence and abundance of uncultivated Megasphaera-like bacteria in the human vaginal environment.. Appl Environ Microbiol.

