# Supplementary figures and images for: Bacterial Communities in Women with Bacterial Vaginosis: High Resolution Phylogenetic Analyses Reveal Relationships of Microbiota to Clinical Criteria
Source: PLoS One. 2012 Jun 18;7(6):e37818. doi: 10.1371/journal.pone.0037818 (PMC3377712; doi:10.1371/journal.pone.0037818)

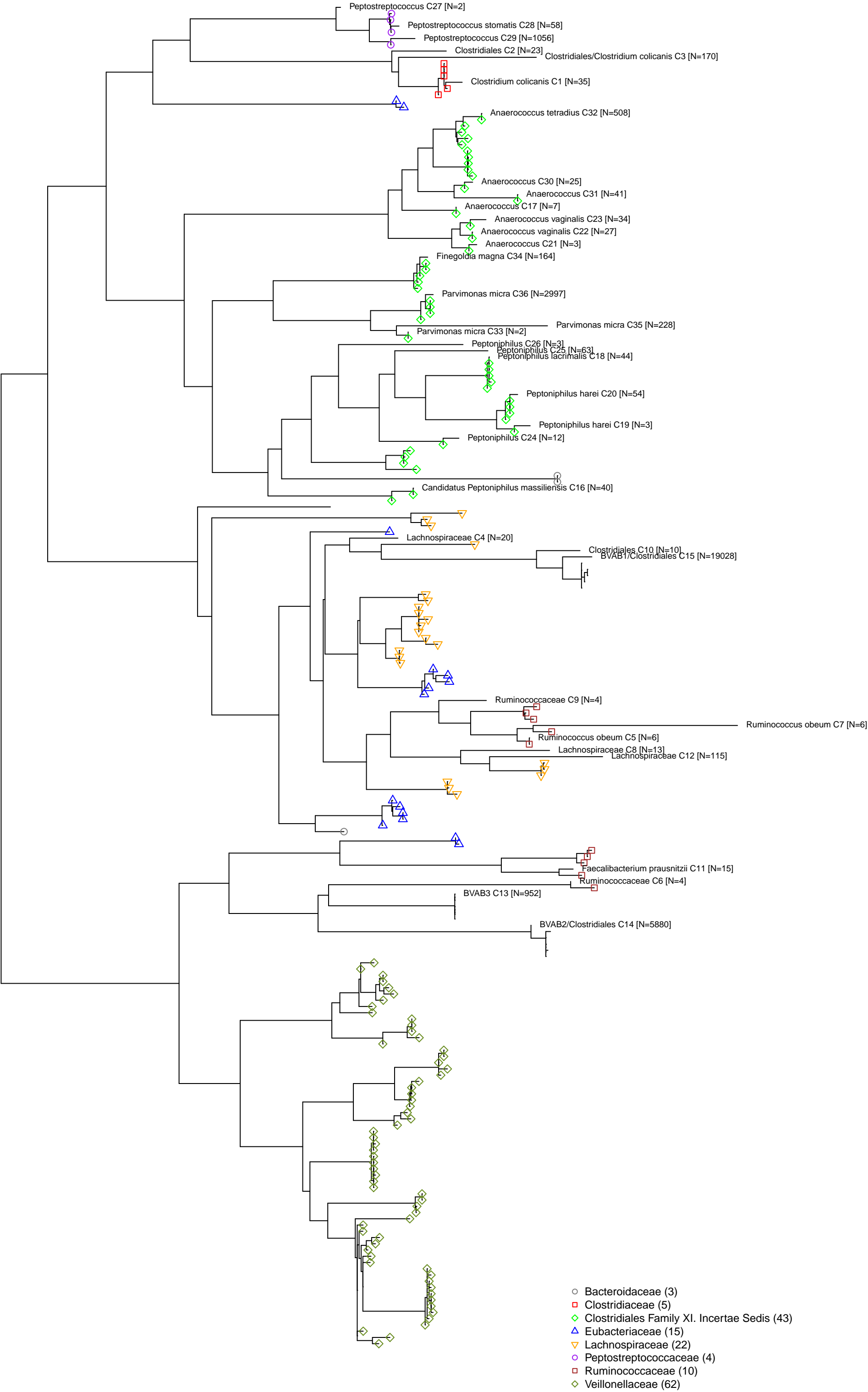

Supplement: Figure S1 — Clusters of sequence reads in the Clostridiales Order. All pyrosequencing reads classified as belonging to the Clostridiales Order were submitted to a phylogenetic approach after placement on the reference tree. A cluster number is indicated after each taxon name. Clusters were labeled only if the sequence reads originated from at least two different subjects. The number of reads in a cluster present in the entire data set is also shown. Symbols denote the family level classification for each taxon. (PDF) [file pone.0037818.s001.pdf]

Supplementary Figure 3A

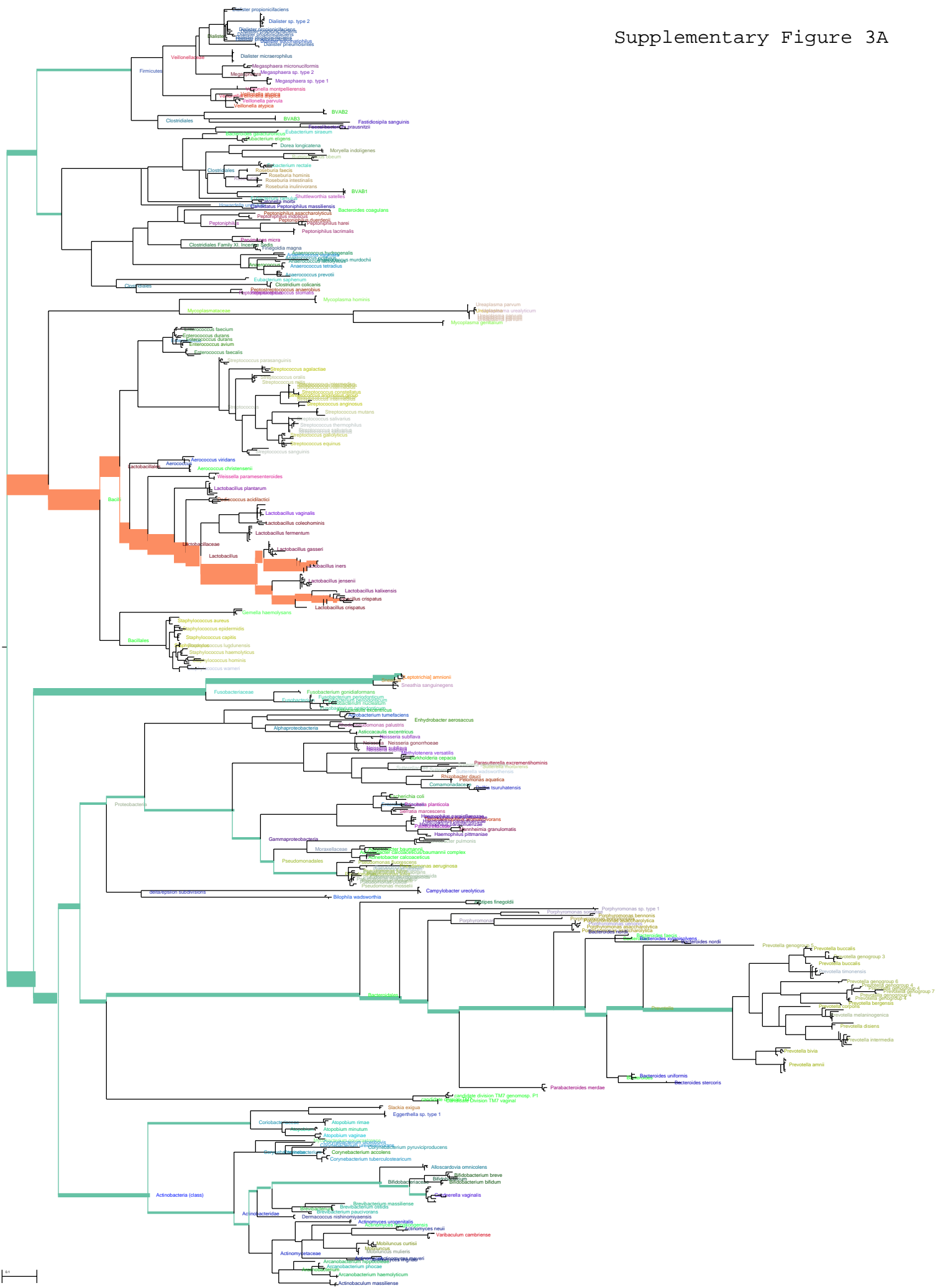

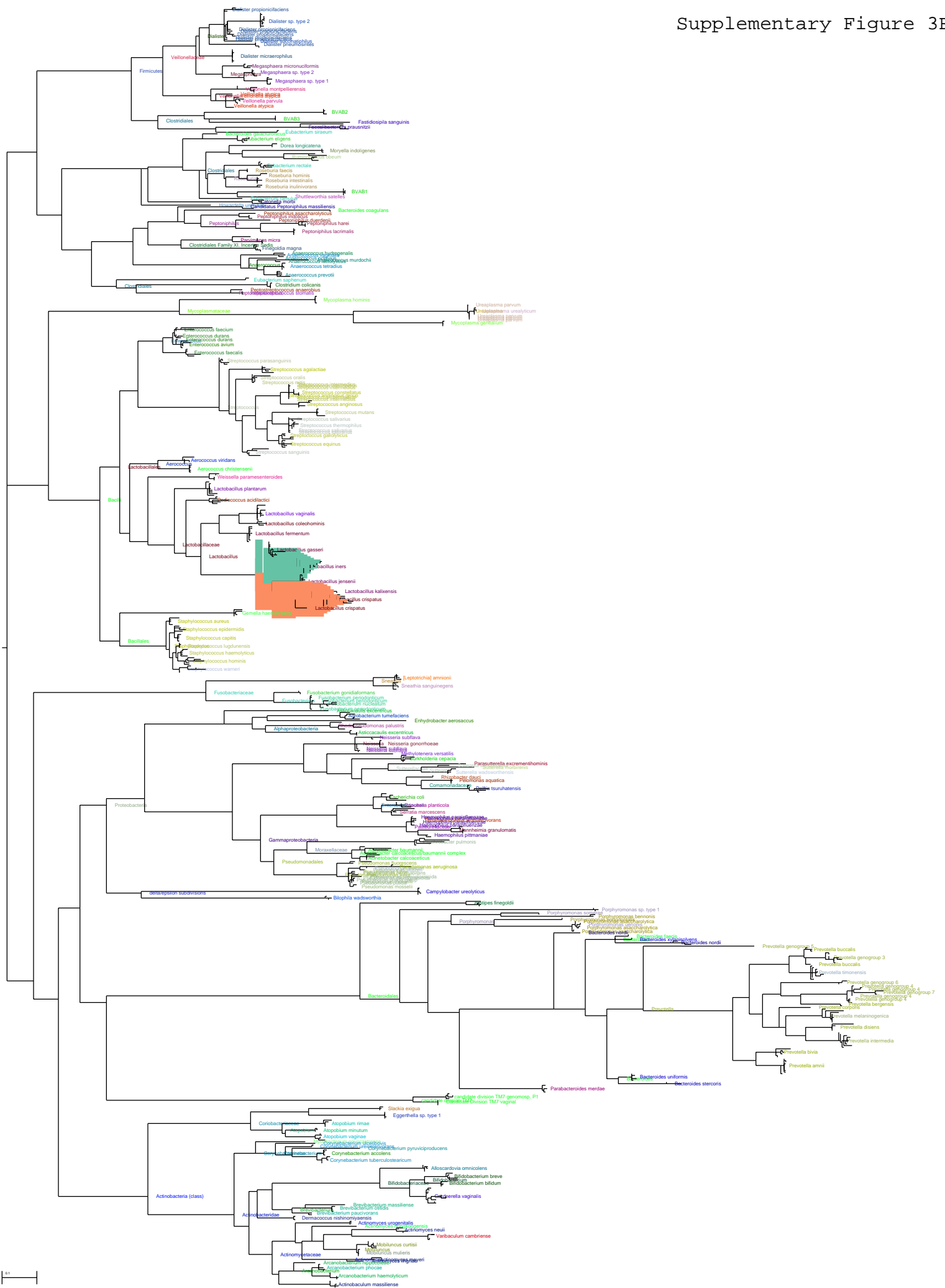

Supplement: Figure S3 — Edge principal component vectors projected on to the phylogenetic tree. These vectors are indexed by edges of the reference tree, and displayed as colored and thickened edges. The thickness of an edge is proportional to its weight in the principal component vector; positive coefficients marked with orange and negative are marked in green. The first principal component (S3A), with 59% of the variance, has all positive coefficients on edges leading to the Lactobacillus clade. The second principal component (S3B), with 17% of the variance, gives a positive coefficient to the Lactobacillus crispatus clade and a negative coefficient to the Lactobacillus iners clade. These tree diagrams justify the axis labels given to Figure 3 in the main text. (PDF) [file pone.0037818.s003.pdf]
