# Supplementary material for: Bacterial Communities in Women with Bacterial Vaginosis: High Resolution Phylogenetic Analyses Reveal Relationships of Microbiota to Clinical Criteria
Source: PLoS One. 2012 Jun 18;7(6):e37818. doi: 10.1371/journal.pone.0037818 (PMC3377712; doi:10.1371/journal.pone.0037818)
Supplement: Figure S2 — Descriptive Statistics. Chao 1 plots comparing species richness and Shannon plots comparing species diversity. Chao Plot 1 (Nugent Score; 0–6 and 7–10): BV is characterized by greater species richness. Chao Plot 2 (Nugent Score; 0–3, 4–6 and 7–10): The median numbers of taxa are similar between those who have Intermediate flora and frank BV. Chao Plot 3 (Amsel’s clinical criteria): Women who are diagnosed for BV by Amsel’s clinical criteria have greater numbers of taxa than those who don’t have BV. Chao Plot 4 (Amsel’s criteria – pH): Higher pH is typically associated with increased species richness. Chao Plot 5 (Amsel’s criteria – Whiff test): Vaginal samples that have an amine odor upon addition of potassium hydroxide are characterized by increased species richness. Chao Plot 6 (Amsel’s criteria – Clue cells): Vaginal samples that have <20% clue cells have similar numbers of taxa when compared to those who have >20% clue cells. Chao Plot 7 (Amsel’s criteria – Vaginal discharge): Women with abnormal vaginal discharge are typically colonized with greater numbers of taxa than those with normal vaginal discharge. Shannon Plot 1 (Nugent Score; 0–6 and 7–10): BV is characterized by increased species diversity. Shannon Plot 2 (Nugent Score; 0–3, 4–6 and 7–10): There is greater species diversity in those who have Intermediate flora (4–6) and frank BV (7–10) when compared with women without BV (0–3). Shannon Plot 3 (Amsel’s clinical criteria): Women who are diagnosed for BV by Amsel’s clinical criteria have greater species diversity than those who don’t have BV. Shannon Plot 4 (Amsel’s criteria – pH): Higher pH is typically associated with increased species diversity. All women with a pH of 4.5 and less were BV negative. Shannon Plot 5 (Amsel’s criteria – Whiff test): Vaginal samples that have an amine odor upon addition of potassium hydroxide are characterized by increased species diversity. Shannon Plot 6 (Amsel’s criteria – Clue cells): Vaginal samples that have clue ce [file pone.0037818.s002.pdf]

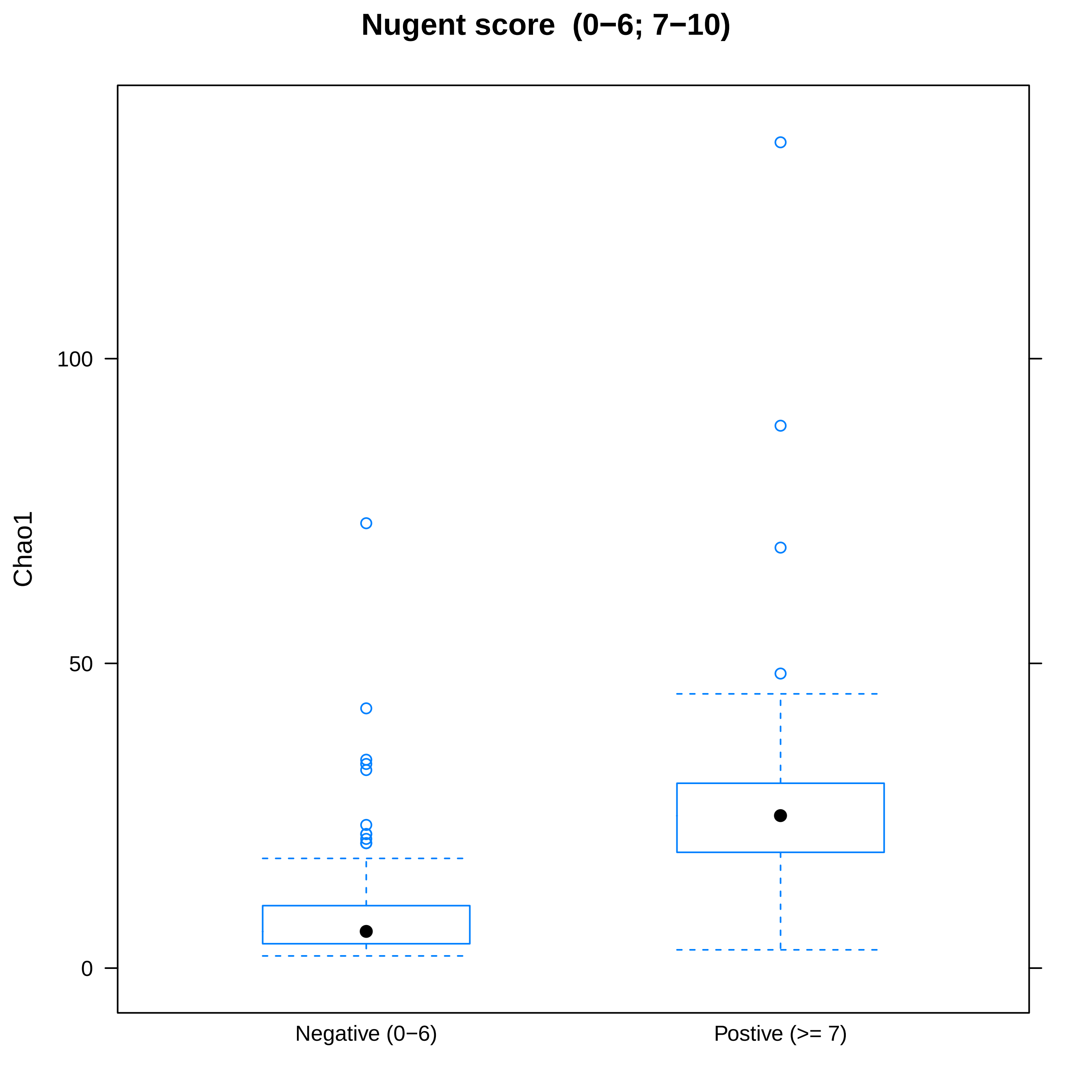

Nugent score (0−6; 7−10)
Chao1
100
50
0
Negative (0−6)
Postive (>= 7)

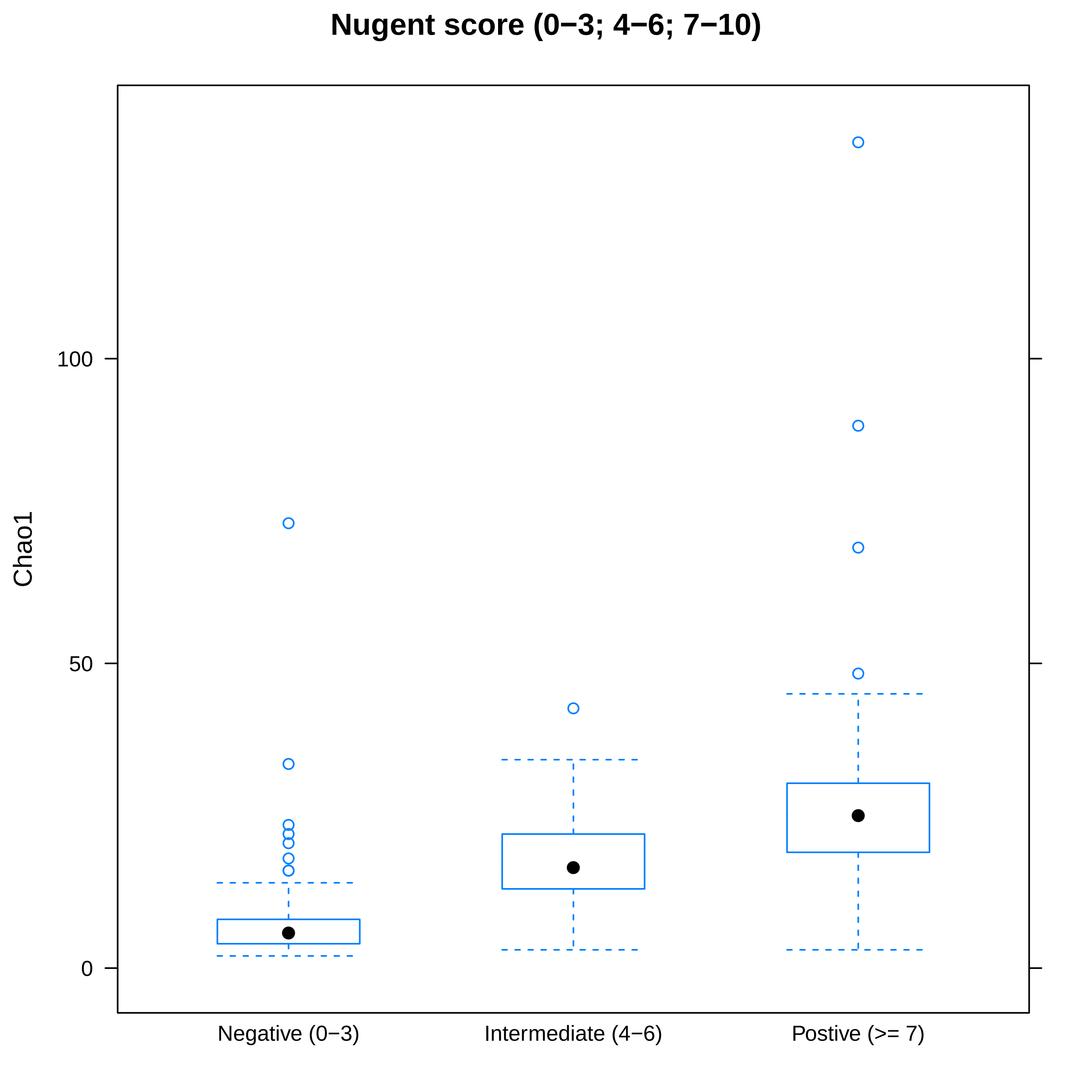

Nugent score (0−3; 4−6; 7−10)
Chao1
100
50
0
Negative (0−3)
Intermediate (4−6)
Postive (>= 7)

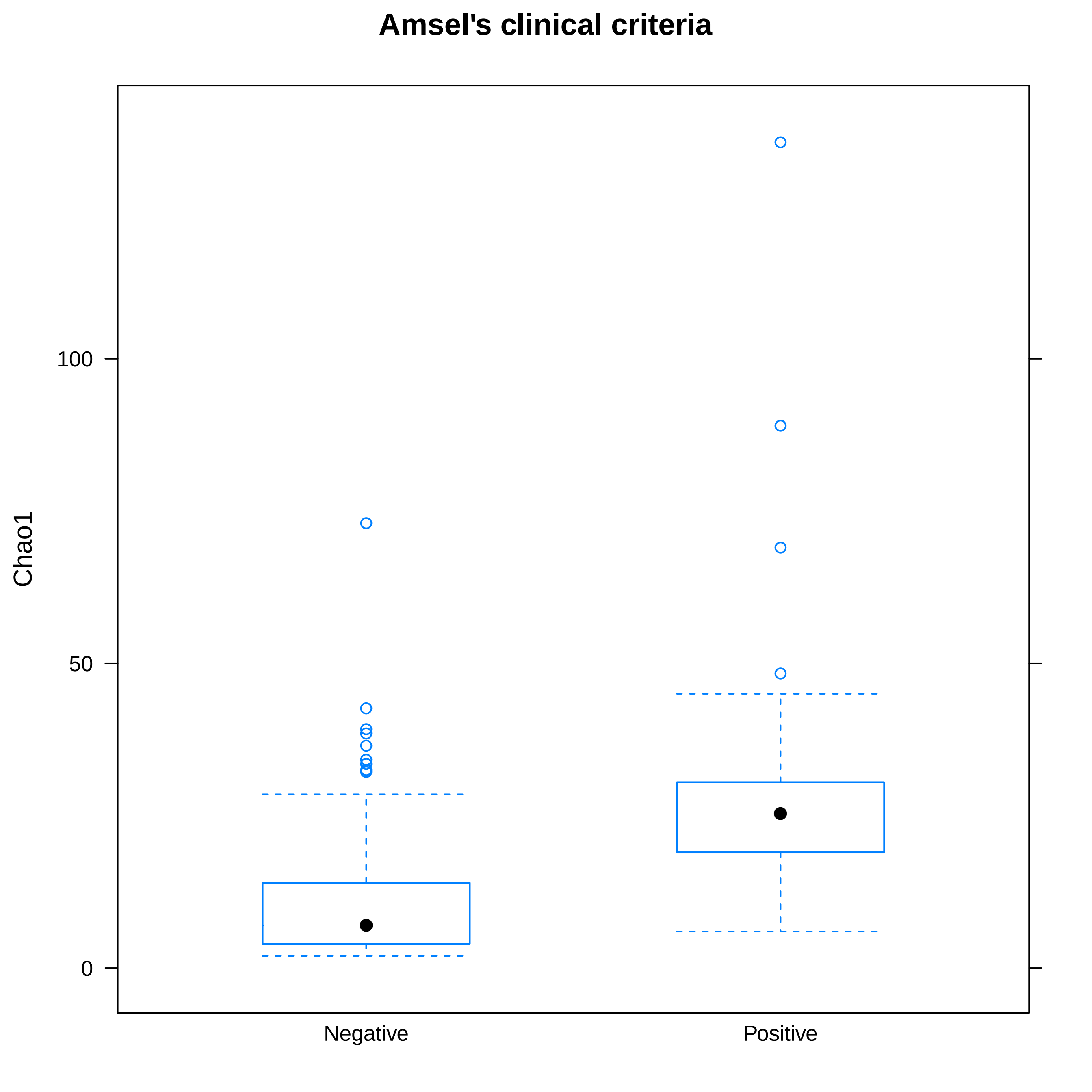

Amsel's clinical criteria
Chao1
100
50
0
Negative
Positive

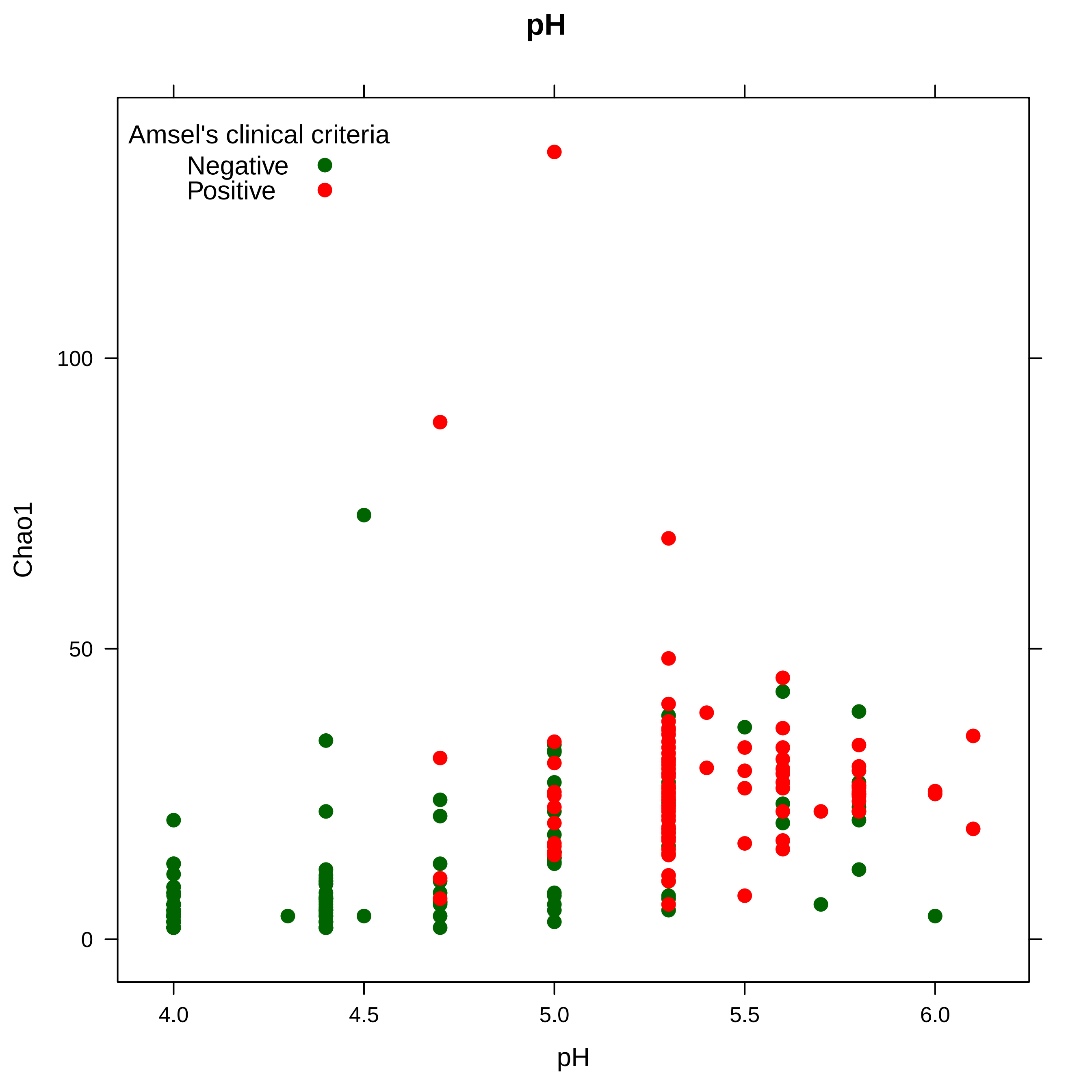
pH
Amsel's clinical criteria
Negative
Positive
Chao1
0
50
100
4.0
4.5
5.0
5.5
6.0
pH

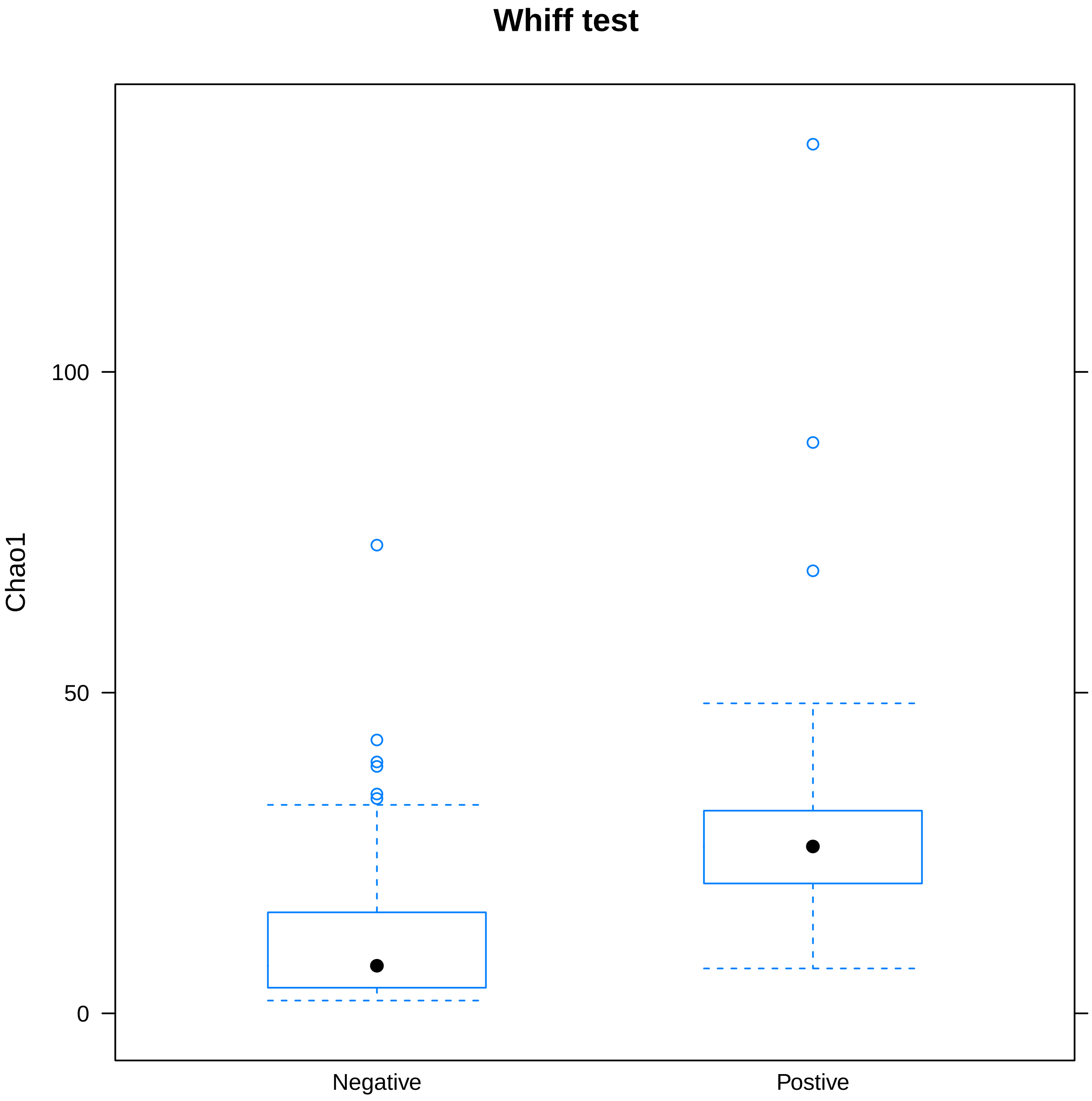

Whiff test
Chao1
100
50
0
Negative
Postive

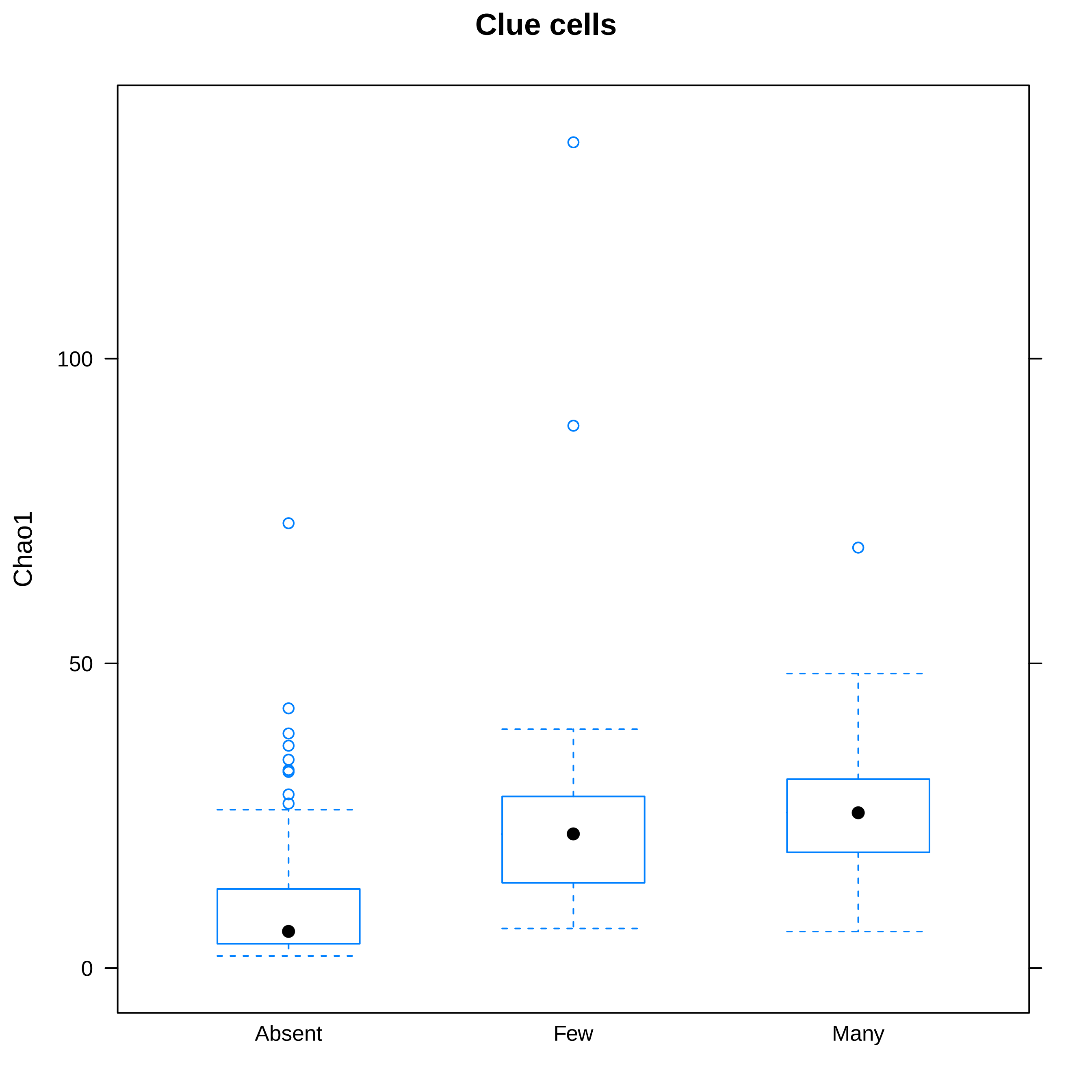

Clue cells
Chao1
100
50
0
Absent
Few
Many

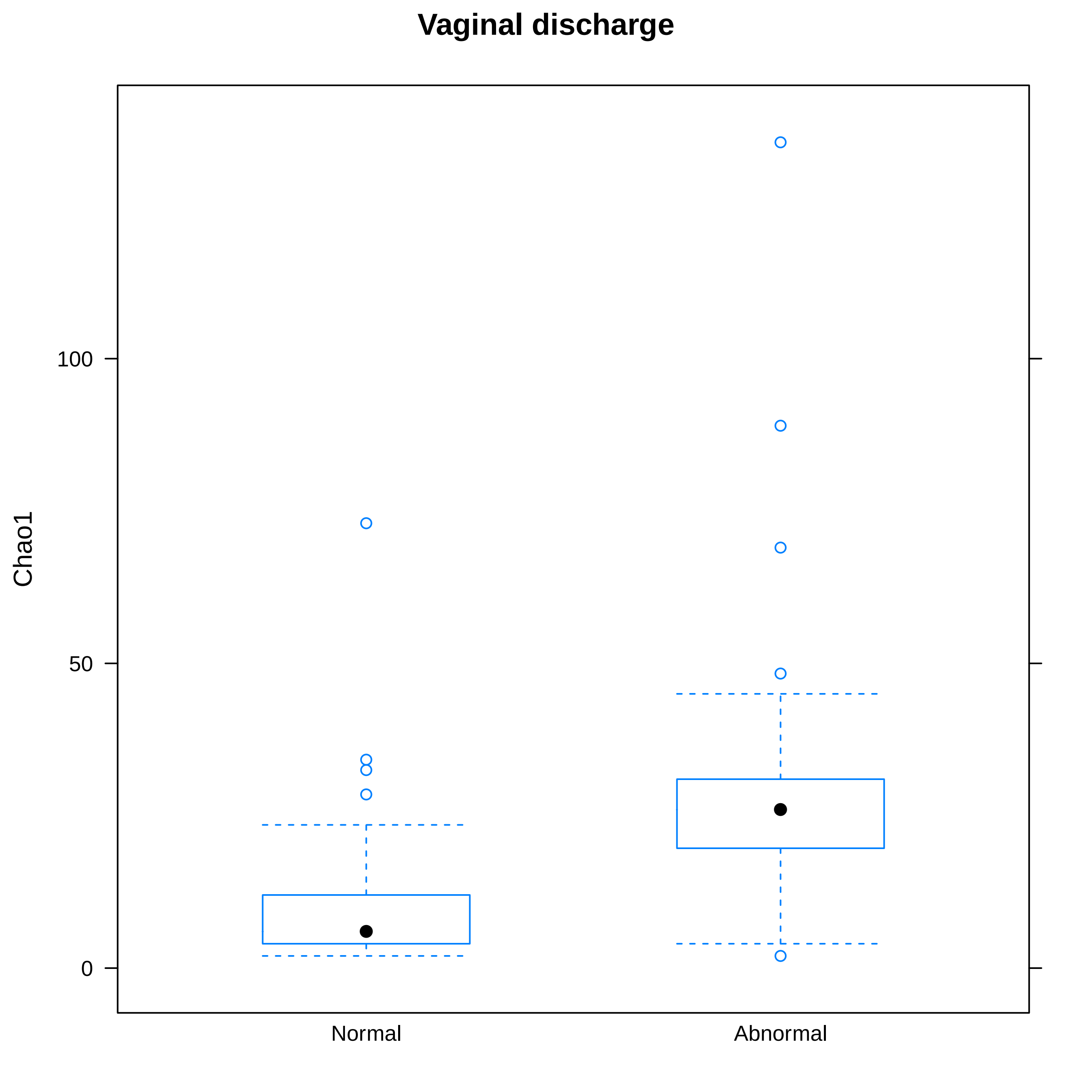

Vaginal discharge
Chao1
100
50
0
Normal
Abnormal

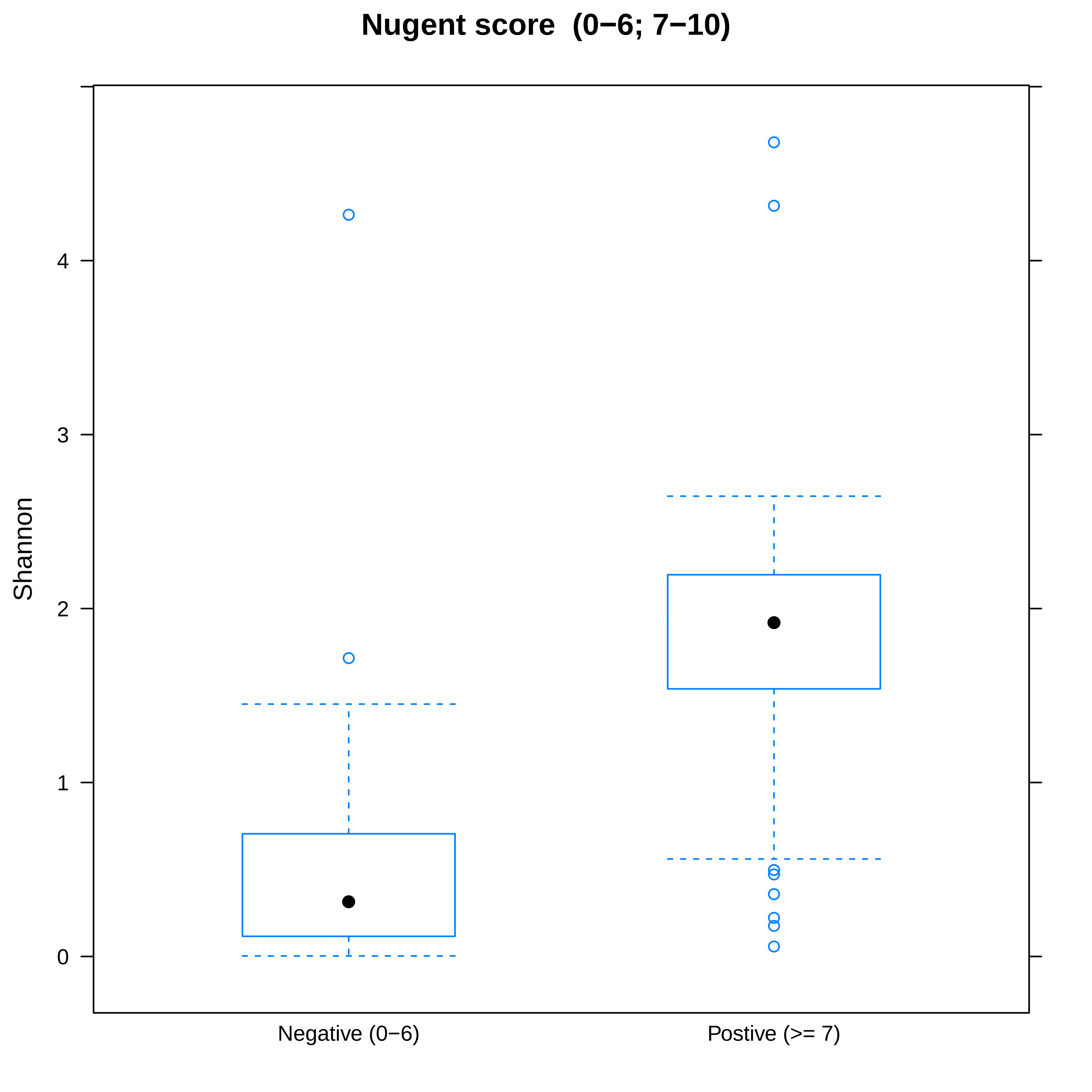
Nugent score (0−6; 7−10)
Shannon
0
1
2
3
4
Negative (0−6)
Postive (>= 7)

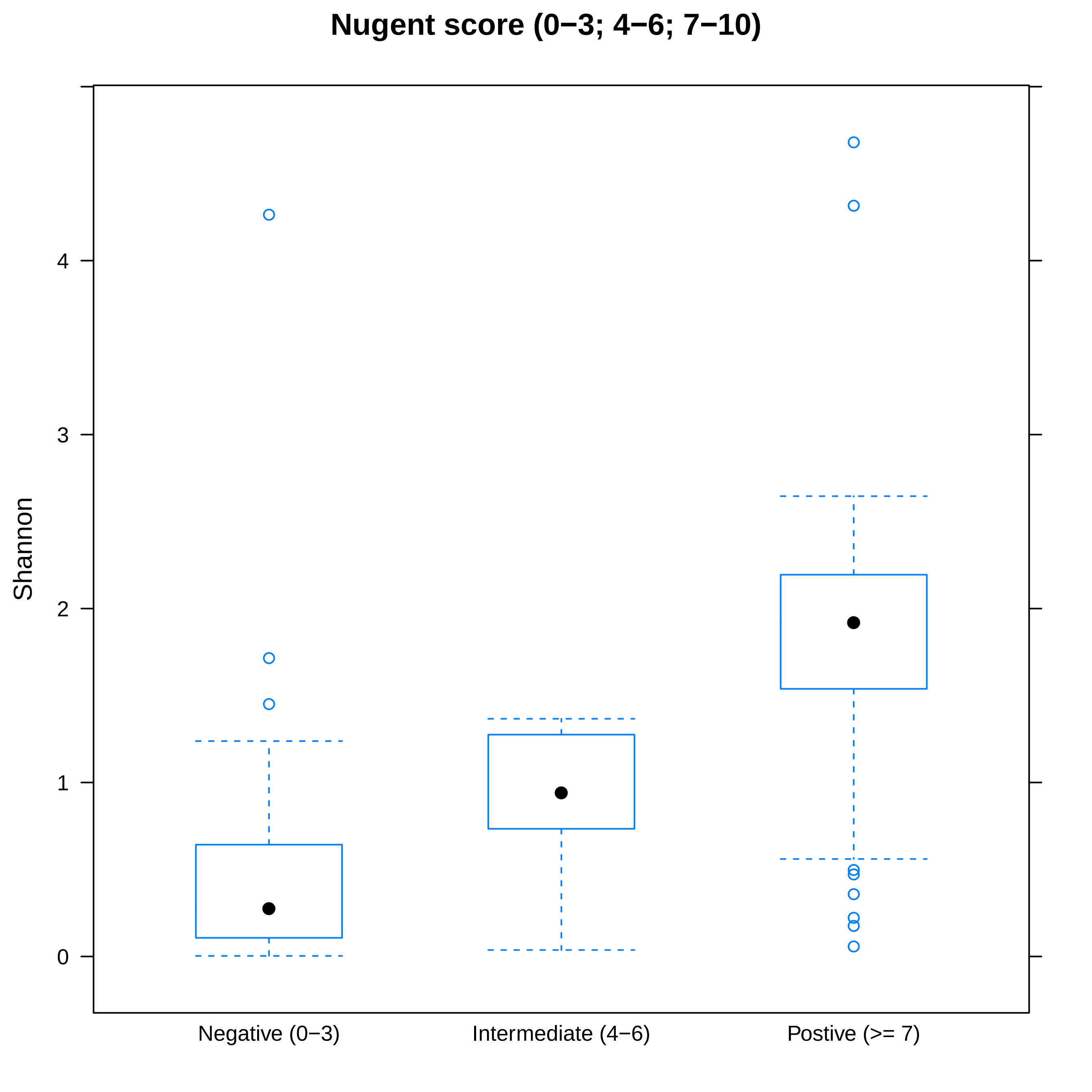
Nugent score (0−3; 4−6; 7−10)
Shannon
0
1
2
3
4
Negative (0−3)
Intermediate (4−6)
Postive (>= 7)

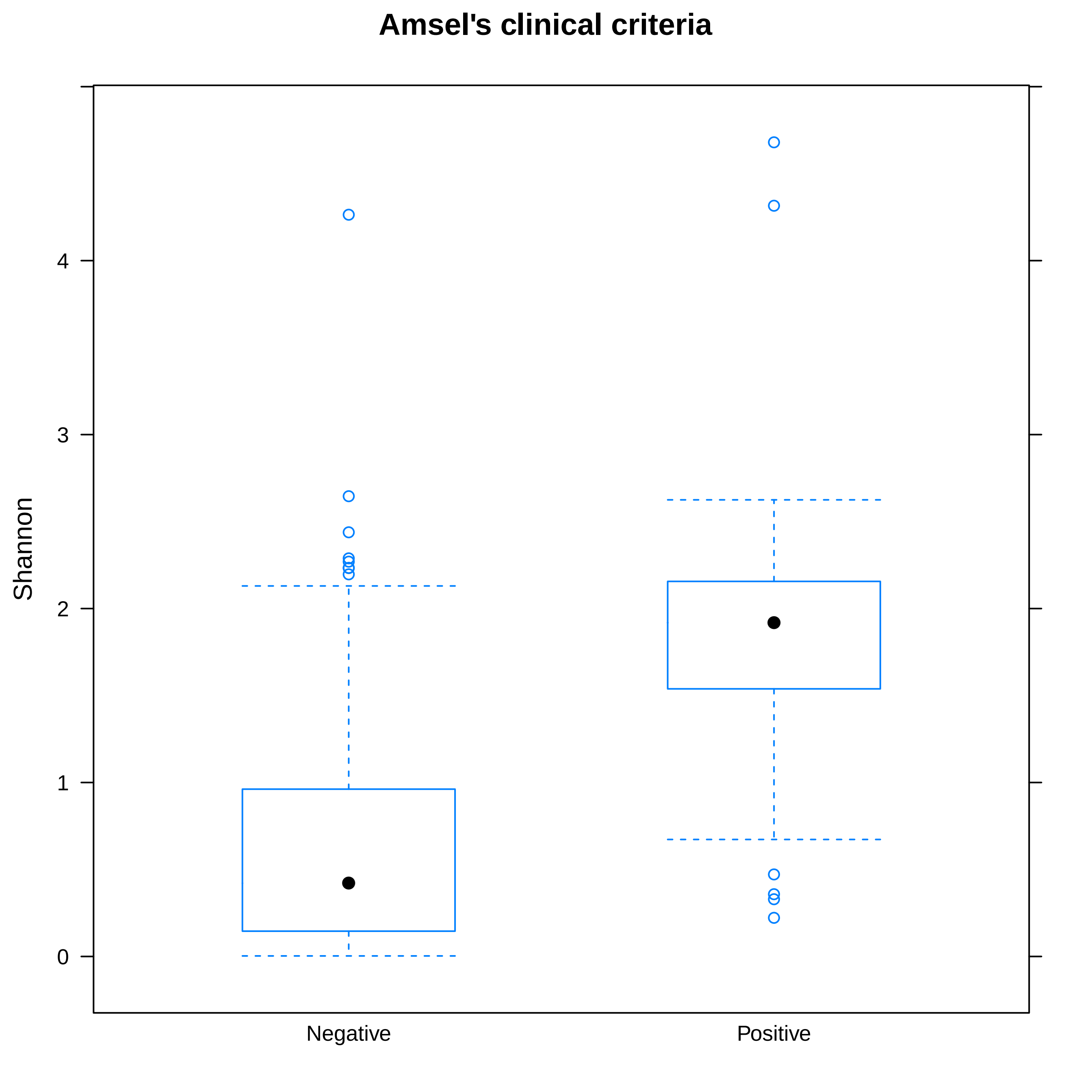
Amsel's clinical criteria
Shannon
0
1
2
3
4
Negative
Positive

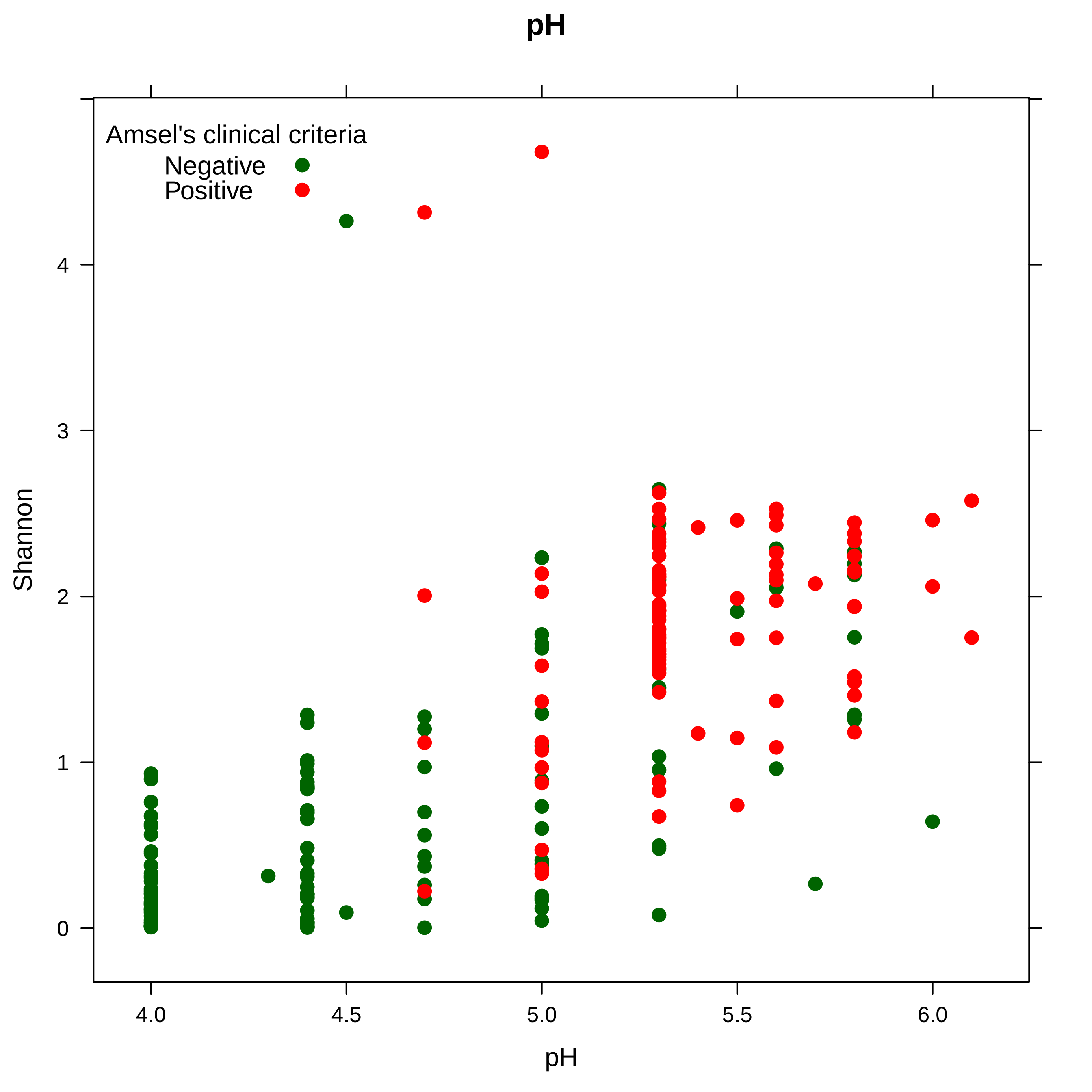
pH
Amsel's clinical criteria
Negative
Positive
Shannon
0
1
2
3
4
4.0
4.5
5.0
5.5
6.0
pH

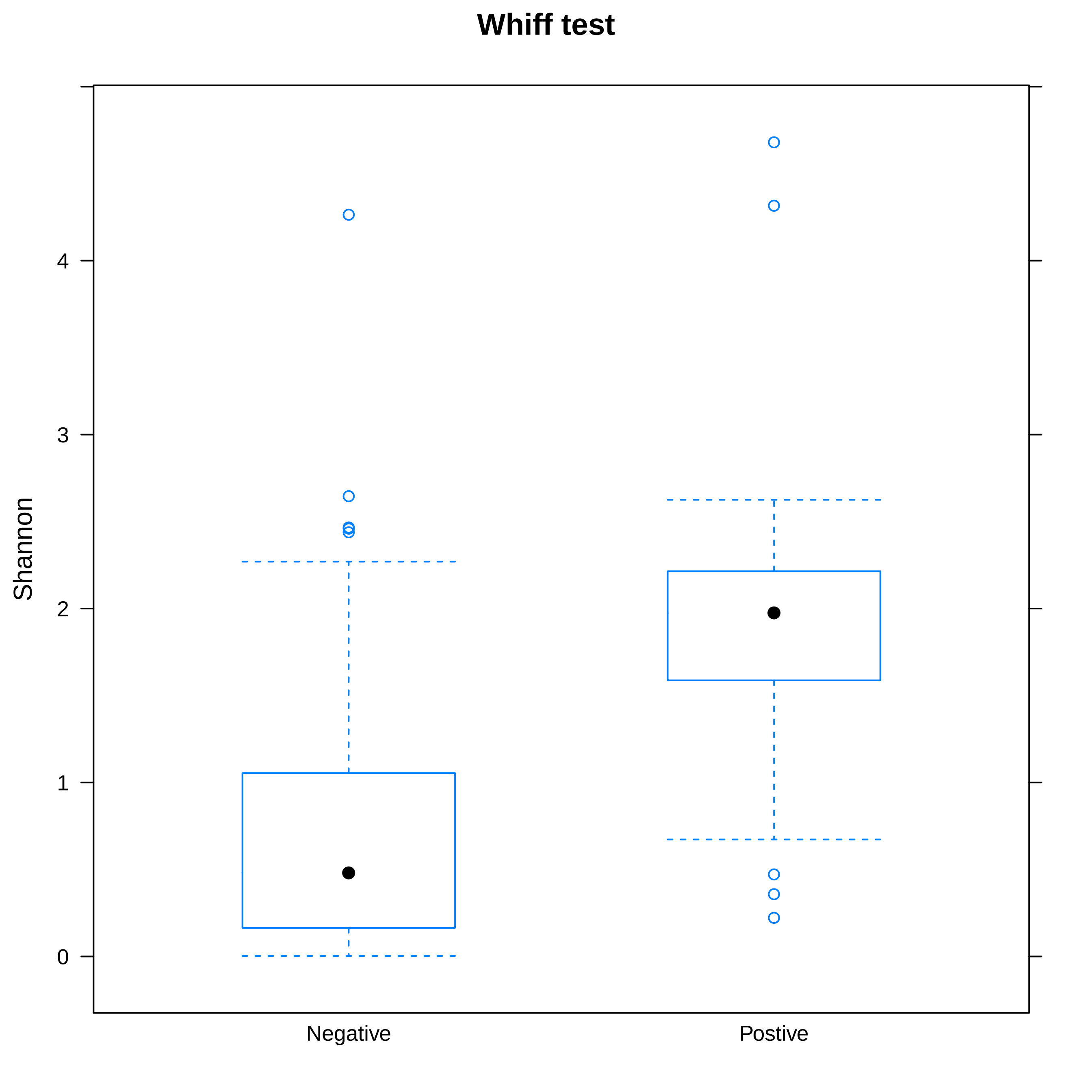
Whiff test
Shannon
0
1
2
3
4
Negative
Postive

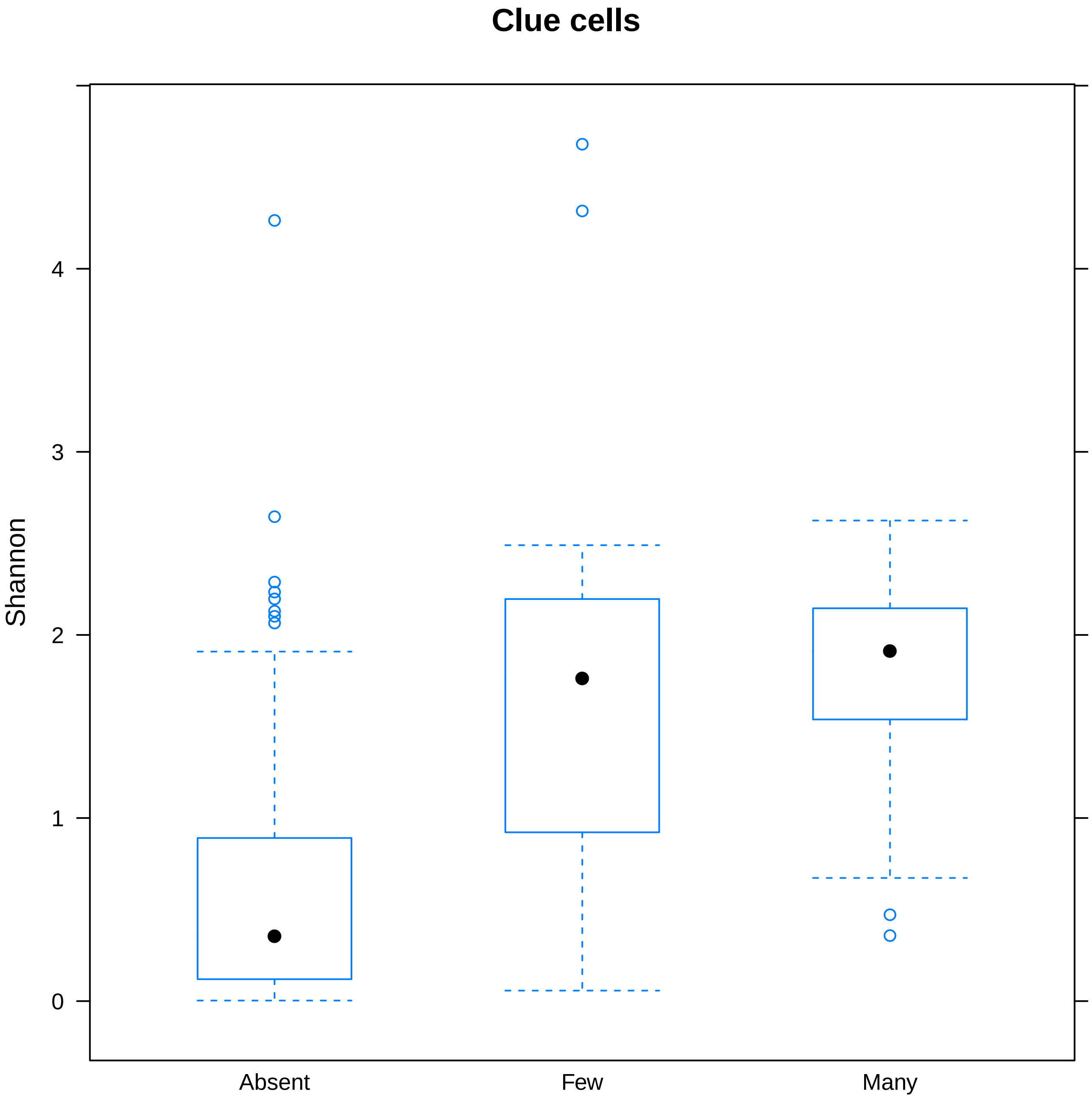

Clue cells
Shannon
0
1
2
3
4
Absent
Few
Many

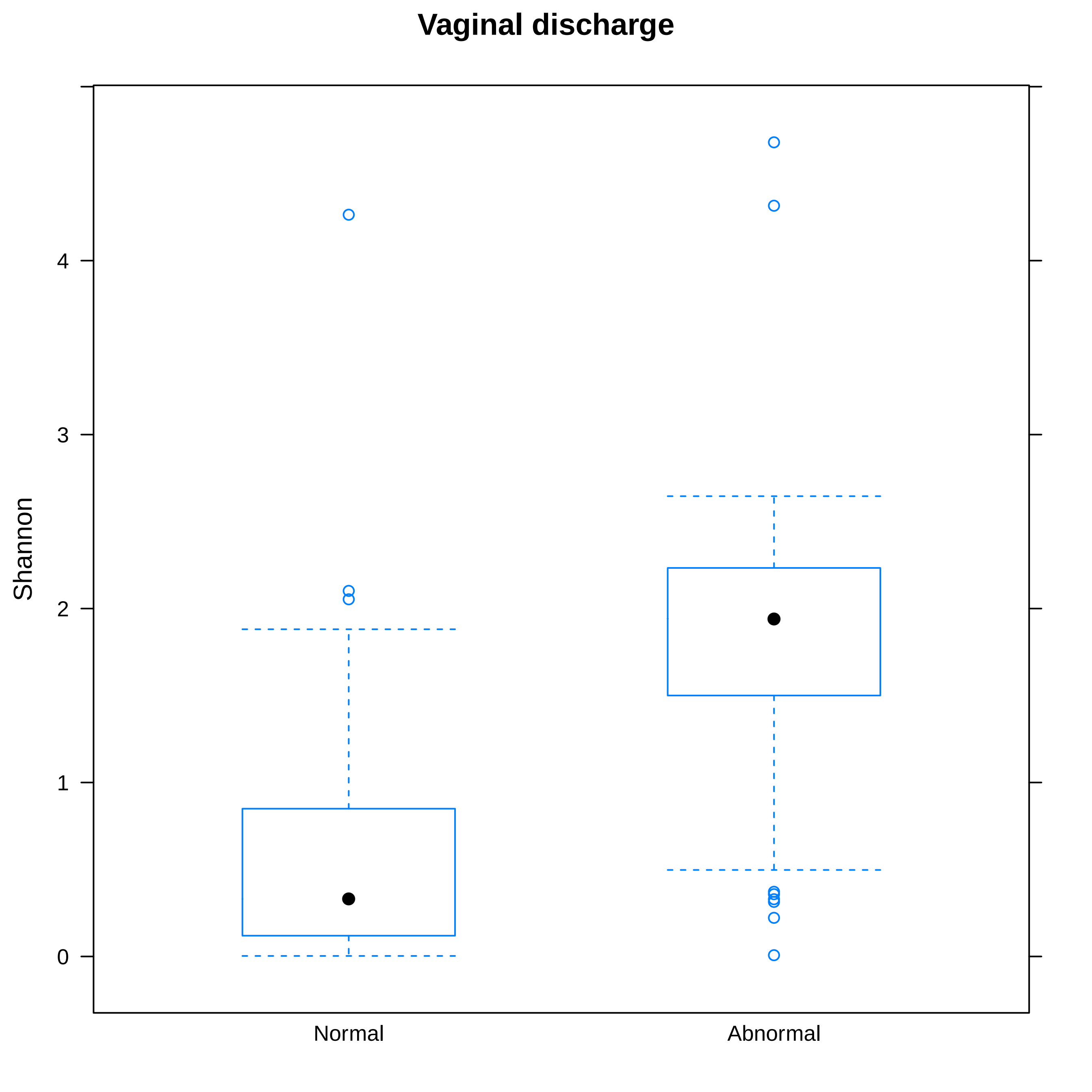
Vaginal discharge
Shannon
0
1
2
3
4
Normal
Abnormal
