# Supplementary material for: Bacterial Communities in Women with Bacterial Vaginosis: High Resolution Phylogenetic Analyses Reveal Relationships of Microbiota to Clinical Criteria
Source: PLoS One. 2012 Jun 18;7(6):e37818. doi: 10.1371/journal.pone.0037818 (PMC3377712; doi:10.1371/journal.pone.0037818)
Supplement: Text S1 — Classifier Validation. (PDF) [file pone.0037818.s013.pdf]

Supplementary Material

Classifier validation: Taxonomic assignment for *Lactobacillus* spp. was systematically evaluated by assembling a validation set of 16S rRNA gene sequences representing a subset of species belonging to this genus consisting of all available high-quality, near-full-length records available from the RDP. The reference set comprised *Lactobacillus* species that have been shown to be present in the human vagina including *L.crispatus*, *L. iners*, *L. gasseri*, *L. jensenii*, and *L. vaginalis* [1,2,3,4]. One representative sequence from each group of identical sequences was retained for validation. Sequences used for validation also met the same distance criteria as described for the reference set creation in the main text to avoid introducing mislabeled records into the analysis. Sequences were trimmed to span the same 16S rRNA region as the query reads. The classification outputs summarizing the placement of the validation set of *Lactobacillus* sequences are provided. *L. iners, L. jensenii, L. gasseri*, and *L. vaginalis* displayed 100% sensitivity. *L. crispatus* had a sensitivity of 94% with 3% sequences classified at genus level and 3% sequences as *L. fermentum*. *L. acidophilus, L. helveticus*, and *L. ultunensis* were classified as *L. crispatus*. We did not include these 3 lactobacilli in the reference tree as many studies have shown that they are not dominant in the human vagina [1,2,4,5,6,7]. Likewise, *L. johnsonii* was classified as *L. gasseri* and was not included in the reference set. It is entirely possible that low numbers of these bacteria may be present in our dataset and our classification approach would likely place them as *L. crispatus* or *L. gasseri* which is a limitation of this study. The use of longer length sequences will facilitate better distinction between closely related species and the 454 titanium chemistry now offers read lengths of up to 450 bp.

Tally of query sequences:

Lactobacillus **species**	**Number of Sequences**
Lactobacillus coleohominis	3
Lactobacillus crispatus	34
Lactobacillus fermentum	284
Lactobacillus gasseri	21
Lactobacillus iners	2
Lactobacillus jensenii	11
Lactobacillus plantarum	488
Lactobacillus vaginalis	9

In the tables below, rows are labeled with the names of query sequences as provided by RDP and columns are labeled with the classification from the pipeline. Species names marked by an asterisk (*) are not represented in the classification pipeline.

In **Table 1**, results are restricted by the label on the input sequence to species belonging to the rows shown (demonstrating sensitivity). In **Table 2**, results are restricted by classification result (demonstrating specificity).

References:

1. Fredricks DN, Fiedler TL, Marrazzo JM (2005) Molecular identification of bacteria associated with bacterial vaginosis. New England Journal of Medicine 353: 1899-1911.
2. Hyman RW, Fukushima M, Diamond L, Kumm J, Giudice LC, et al. (2005) Microbes on the human vaginal epithelium. Proceedings of the National Academy of Sciences of the United States of America 102: 7952-7957.
3. Oakley BB, Fiedler TL, Marrazzo JM, Fredricks DN (2008) The Diversity of Human Vaginal Bacterial Communities and their Association with Clinically-Defined Bacterial Vaginosis. Appl Environ Microbiol.
4. Ravel J, Gajer P, Abdo Z, Schneider GM, Koenig SS, et al. (2010) Microbes and Health Sackler Colloquium: Vaginal microbiome of reproductive-age women. Proc Natl Acad Sci U S A
5. Gustafsson RJ, Ahrne A, Jeppsson B, Benoni C, Olsson C, et al. (2011) The Lactobacillus flora in vagina and rectum of fertile and postmenopausal healthy Swedish women. BMC Women's Health 11: 17
6. Schellenberg JJ, Links MG, Hill JE, Dumonceaux TJ, Kimani J, et al. (2011) Molecular Definition of Vaginal Microbiota in East African Commercial Sex Workers. Applied and Environmental Microbiology 77: 4066-4074.

7. Spear GT, Gilbert D, Landay AL, Zariffard R, French AL, et al. (2011) Pyrosequencing of the Genital Microbiotas of HIV-Seropositive and -Seronegative Women Reveals Lactobacillus iners as the Predominant Lactobacillus Species. Applied and Environmental Microbiology 77: 378-381.

Table 1

species_name	Enterococcus	Lactobacillaceae	Lactobacillales	Lactobacillus	coleohominis	crispatus
Lactobacillus coleohominis (3)	0	0	0	0	3	0
Lactobacillus crispatus (34)	0	0	0	1	0	32
Lactobacillus fermentum (284)	0	0	0	1	0	0
Lactobacillus gasseri (21)	0	0	0	0	0	0
Lactobacillus iners (2)	0	0	0	0	0	0
Lactobacillus jensenii (11)	0	0	0	0	0	0
Lactobacillus plantarum (488)	1	2	1	3	0	0
Lactobacillus vaginalis (9)	0	0	0	0	0	0

	classif_name						
species_name	fermentum	gasseri	iners	jensenii	plantarum	vaginalis	Moraxellaceae
Lactobacillus coleohominis (3)	0	0	0	0	0	0	0
Lactobacillus crispatus (34)	1	0	0	0	0	0	0
Lactobacillus fermentum (284)	283	0	0	0	0	0	0
Lactobacillus gasseri (21)	0	21	0	0	0	0	0
Lactobacillus iners (2)	0	0	2	0	0	0	0
Lactobacillus jensenii (11)	0	0	0	11	0	0	0
Lactobacillus plantarum (488)	0	0	0	0	480	0	1
Lactobacillus vaginalis (9)	0	0	0	0	0	9	0

Table 2

species_name	Lactobacillaceae	Lactobacillus	coleohominis	crispatus	fermentum	gasseri	iners
Lactobacillus coleohominis (3)	0	0	3	0	0	0	0
Lactobacillus crispatus (34)	0	1	0	32	1	0	0
Lactobacillus fermentum (284)	0	1	0	0	283	0	0
Lactobacillus gasseri (21)	0	0	0	0	0	21	0
Lactobacillus iners (2)	0	0	0	0	0	0	2
Lactobacillus jensenii (11)	0	0	0	0	0	0	0
Lactobacillus plantarum (488)	2	3	0	0	0	0	0
Lactobacillus vaginalis (9)	0	0	0	0	0	0	0
* Lactobacillus acidophilus (20)	0	0	0	20	0	0	0
* Lactobacillus amylolyticus (1)	0	1	0	0	0	0	0
* Lactobacillus antri (1)	0	0	0	0	0	0	0
* Lactobacillus brevis (133)	0	133	0	0	0	0	0
* Lactobacillus buchneri (17)	16	0	0	0	0	0	0
* Lactobacillus helveticus (647)	0	0	0	647	0	0	0
* Lactobacillus hilgardii (14)	0	0	0	0	0	0	0
* Lactobacillus johnsonii (10)	0	0	0	0	0	10	0
* Lactobacillus reuteri (65)	0	1	0	0	0	0	0
* Lactobacillus rhamnosus (55)	1	0	0	0	0	0	0
* Lactobacillus ultunensis (2)	0	0	0	2	0	0	0

species_name	jensenii	plantarum	vaginalis
Lactobacillus coleohominis (3)	0	0	0
Lactobacillus crispatus (34)	0	0	0
Lactobacillus fermentum (284)	0	0	0
Lactobacillus gasseri (21)	0	0	0
Lactobacillus iners (2)	0	0	0
Lactobacillus jensenii (11)	11	0	0
Lactobacillus plantarum (488)	0	480	0
Lactobacillus vaginalis (9)	0	0	9
* Lactobacillus acidophilus (20)	0	0	0
* Lactobacillus amylolyticus (1)	0	0	0
* Lactobacillus antri (1)	0	0	1
* Lactobacillus brevis (133)	0	0	0
* Lactobacillus buchneri (17)	0	0	1
* Lactobacillus helveticus (647)	0	0	0
* Lactobacillus hilgardii (14)	0	0	14
* Lactobacillus johnsonii (10)	0	0	0
* Lactobacillus reuteri (65)	0	0	64
* Lactobacillus rhamnosus (55)	0	0	0
* Lactobacillus ultunensis (2)	0	0	0
